# Specificities and Commonalities of Carbapenemase-Producing Escherichia coli Isolated in France from 2012 to 2015

**DOI:** 10.1128/msystems.01169-21

**Published:** 2022-01-11

**Authors:** Rafael Patiño-Navarrete, Isabelle Rosinski-Chupin, Nicolas Cabanel, Pengdbamba Dieudonné Zongo, Mélanie Héry, Saoussen Oueslati, Delphine Girlich, Laurent Dortet, Rémy A. Bonnin, Thierry Naas, Philippe Glaser

**Affiliations:** a Unité EERA, Institut Pasteur, APHP, Université Paris-Saclay, Paris, France; b UMR3525, CNRS, Université de Paris, Paris, France; c Sorbonne Université, Paris, France; d Team “Resist” INSERM U1184 “Immunology of Viral, Auto-Immune, Hematological and Bacterial diseases (IMVA-HB)”, Le Kremlin-Bicêtre, France; e Department of Bacteriology-Hygiene, Bicêtre Hospital, APHP, Le Kremlin-Bicêtre, France; f Associated French National Reference Center for Antibiotic Resistance, Le Kremlin-Bicêtre, France; University of Illinois at Chicago

**Keywords:** drug resistance evolution, emergence, genomics, multidrug resistance, surveillance studies

## Abstract

Carbapenemase-producing Escherichia coli (CP-*Ec*) represents a major public health threat with a risk of dissemination in the community as has occurred for lineages producing extended-spectrum β-lactamases. To characterize the extent of CP-*Ec* spread in France, isolates from screening and infection samples received at the French National Reference Center (F-NRC) laboratory for carbapenemase-producing *Enterobacterales* were investigated. A total of 691 CP-*Ec* isolates collected between 2012 and 2015 and 22 isolates collected before 2012 were fully sequenced. Analysis of their genome sequences revealed some disseminating multidrug-resistant (MDR) lineages frequently acquiring diverse carbapenemase genes mainly belonging to clonal complex 23 (CC23) (sequence type 410 [ST410]) and CC10 (ST10 and ST167) and sporadic isolates, including rare ST131 isolates (*n* = 17). However, the most represented sequence type (ST) was ST38 (*n* = 92) with four disseminated lineages carrying *bla*_OXA-48-like_ genes inserted in the chromosome. Globally, the most frequent carbapenemase gene (*n* = 457) was *bla*_OXA-48_. It was also less frequently associated with MDR isolates being the only resistance gene in 119 isolates. Thus, outside the ST38 clades, its acquisition was frequently sporadic with no sign of dissemination, reflecting the circulation of the IncL plasmid pOXA-48 in France and its high frequency of conjugation. In contrast, *bla*_OXA-181_ and *bla*_NDM_ genes were often associated with the evolution of MDR E. coli lineages characterized by mutations in *ftsI* and *ompC.*

**IMPORTANCE** Carbapenemase-producing Escherichia coli (CP-*Ec*) might be difficult to detect, as MICs can be very low. However, their absolute number and their proportion among carbapenem-resistant *Enterobacterales* have been increasing, as reported by WHO and national surveillance programs. This suggests a still largely uncharacterized community spread of these isolates. Here, we have characterized the diversity and evolution of CP-*Ec* isolated in France before 2016. We show that carbapenemase genes are associated with a wide variety of E. coli genomic backgrounds and a small number of dominant phylogenetic lineages. In a significant proportion of CP-*Ec*, the most frequent carbapenemase gene *bla*_OXA-48_, was detected in isolates lacking any other resistance gene, reflecting the dissemination of pOXA-48 plasmids, likely in the absence of any antibiotic pressure. In contrast, carbapenemase gene transfer may also occur in multidrug-resistant E. coli, ultimately giving rise to at-risk lineages encoding carbapenemases with a high potential of dissemination.

## INTRODUCTION

Escherichia coli is one of the first causes of diverse bacterial infections in the community and in the hospital. In particular, it is the most frequent cause of urinary tract infection (UTI), and 50 to 60% of women will suffer at least one UTI during her life (1). Therefore, multidrug resistance (MDR) in E. coli is a major public health issue making E. coli infections more difficult to treat. In addition, as carbapenemase-producing *Enterobacterales* (CPE) are increasingly detected (2, 3) and E. coli is a ubiquitous member of the human gut microbiome, carbapenemase-producing E. coli (CP-*Ec*) is also becoming a major actor for the global dissemination of carbapenemase genes (4).

The emergence and spread of carbapenem-resistant Gram-negative bacteria are mainly linked to the widespread dissemination through horizontal gene transfer (HGT) of mobile genetic elements (MGEs) encoding carbapenemases. These carbapenemases belong to Ambler class A (mainly KPC type), class B (metallo-β-lactamases IMP, VIM, and NDM types) or class D (OXA-48-like enzymes) of β-lactamases (5). The global epidemiology of extended-spectrum β-lactamase (ESBL)-producing E. coli has been characterized through multiple studies, revealing in Western countries the major contribution of the sequence type 131 (ST131) lineage in the high prevalence of *bla*_CTX-M_ family ESBL genes (6). Much less is known with respect to CP-*Ec*.

Since 2012, the French National Reference Center (F-NRC) laboratory for carbapenemase-producing *Enterobacterales* has experienced a steady increase in the number of CP-*Ec* isolates received each year (2). A multilocus sequence typing (MLST) analysis of isolates received in 2012 and 2013 revealed a broad diversity of sequence types (STs), as the 140 analyzed isolates belonged to 50 different STs. However, a few STs were overrepresented (7), such as the ST38 (24 isolates) and ST410 (10 isolates). In that study, only one isolate belonged to ST131, contrasting with the situation in the United Kingdom where ST131 isolates represented 10% of the CP-*Ec* isolates received between 2014 and 2016 by Public Health England (8). On the other hand, a genome-based survey of CPE in the Netherlands between 2014 and 2018 revealed that the 264 analyzed E. coli isolates belonged to 87 different STs, with three dominant lineages, ST38 (*n* = 46), ST167 (*n* = 22), and ST405 (*n* = 16) representing 32% of the isolates (9). F-NRC isolates also showed a predominance of isolates producing OXA-48-like carbapenemases, followed by NDM-producing isolates and suggested clonally related isolates among ST38 OXA-48-producing isolates and ST410 OXA-181-producing isolates, respectively (7).

Whole-genome sequencing (WGS) significantly increases our ability to infer phylogenetic relationships between isolates. By sequencing 50 ST410 CP-*Ec* isolates received by the F-NRC between 2011 and 2015 (10), we showed that 36 (72%) belonged to a newly described ST410 lineage producing OXA-181 (11). We showed that this clade is characterized by mutations in the two porin genes *ompC* and *ompF* leading to a decreased outer membrane permeability to certain β-lactams and by a four-codon duplication (YRIN) in the *ftsI* gene encoding the penicillin binding protein 3 (PBP3) leading to a decreased susceptibility to β-lactams targeting this PBP (10). After a thorough analysis of CP-*Ec* genome sequences from public databases for mutations in these three genes, we proposed that CP-*Ec* followed three different evolutionary trajectories. In some lineages which are enriched in CP-*Ec* isolates and have disseminated globally, acquisition of carbapenem resistance genes might have been facilitated by the mutations in porin genes and in *ftsI*. In ST38, the genetic background and in particular a specific *ompC* allele with reduced permeability to some β-lactams may have similarly facilitated the acquisition of carbapenemase genes. In contrast, other CP-*Ec* isolates, including isolates from ST131, might have occurred sporadically following the acquisition of plasmids encoding carbapenemase and with no clue of dissemination (10).

Here, we thoroughly characterized the diversity of CP*-Ec* circulating in France by sequencing the genomes of almost all isolates received by the F-NRC from its creation in 2012 until 2015 (see Table S1 in the supplemental material). By combining whole-genome phylogeny with the addition of E. coli genome sequences publicly available (Table S2) and tracking mutations in candidate genes, we show that three different situations are encountered. The high transmission potency of the IncL pOXA-48 plasmids has led to a high frequency of OXA-48-producing isolates, often characterized by susceptibility to most non-β-lactam antibiotics. On the other hand, an increasing number of CP-*Ec* lineages, mainly producing OXA-181 and NDM carbapenemases, are observed in France as in other European countries. These lineages are multidrug-resistant (MDR) or extensively drug-resistant (XRD) lineages, they show several mutations in quinolone resistance-determining regions (QRDRs) and are often mutated in *ftsI* and porin genes. Finally, the rapid dissemination of four OXA-48/OXA-244 ST38 lineages might have been favored by the chromosomal integration of the carbapenemase gene.

10.1128/mSystems.01169-21.7 TABLE S1 Main characteristics of the F-NRC CP-*Ec*. Download Table S1, XLSX file, 0.1 MB. Copyright © 2022 Patiño-Navarrete et al. 2022 Patiño-Navarrete et al. https://creativecommons.org/licenses/by/4.0/ This content is distributed under the terms of the Creative Commons Attribution 4.0 International license.

10.1128/mSystems.01169-21.8 TABLE S2 Main characteristics of genome sequences used for phylogenetic reconstructions. Download Table S2, XLSX file, 0.3 MB. Copyright © 2022 Patiño-Navarrete et al. 2022 Patiño-Navarrete et al. https://creativecommons.org/licenses/by/4.0/ This content is distributed under the terms of the Creative Commons Attribution 4.0 International license.

## RESULTS

### CP-*Ec* isolates collected until 2015 by the French National Reference Center.

**FIG 1:**
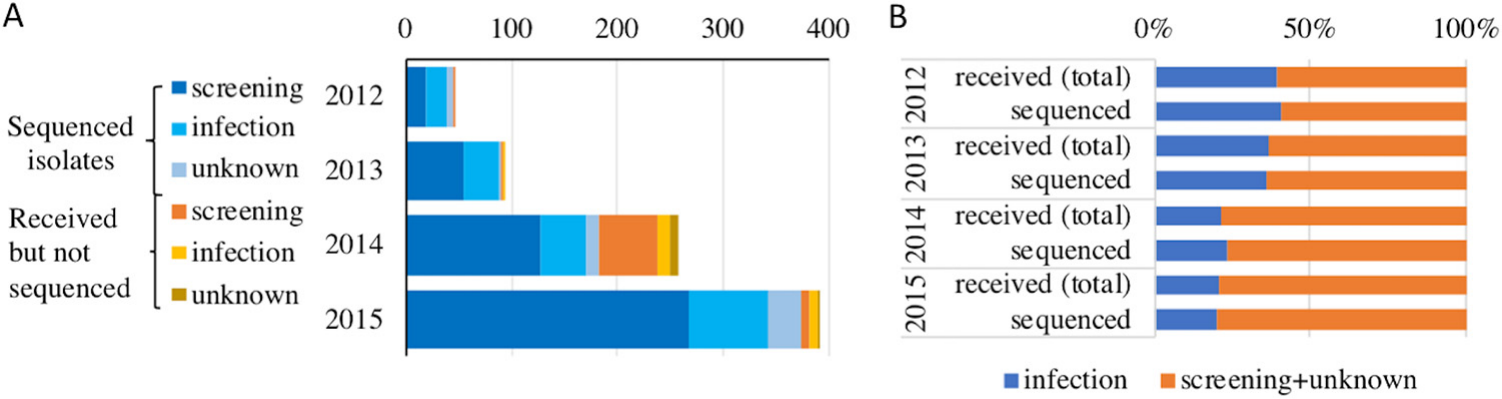
Origin of the isolates received by the F-NRC during the 2012–2015 period. (A) Absolute number of isolates per year from infection, screening, or unknown origins, differentiating between isolates that have or have not been sequenced. (B) Proportions of isolates from infection or from screening or unknown origins showing that isolates from infection origin represent the same proportions among total isolates that have been received by the F-NRC and isolates that have been sequenced.

Isolates analyzed in this work were sent to the F-NRC laboratory on a voluntary basis from private and public clinical laboratories from different parts of France mainly between the years 2012 and 2015. During this period, we encountered a regular increase in the number of isolates received each year (Fig. 1A) corresponding to an increasing number of isolated strains and an increasing frequency of isolates sent to the F-NRC. A total of 713 CP-*Ec* isolates, including 22 isolates collected from 2001 to 2011 were submitted to WGS. The 691 sequenced isolates of the 2012–2015 period represented 87.5% of the 790 CP-*Ec* isolates received by the F-NRC during this period.

The majority of the sequenced isolates (66.5%, 474/713) were from rectal swab screening of patients suspected of carrying CPE (patients repatriated from a hospital abroad, patients who have visited a foreign country within the last 6 months, contact patients of a former carrier, or a previously known carrier), 24% (172/713) were considered to be responsible for infection (isolated from clinical samples), and the source was unknown for 67 isolates (Fig. 1A; see Table S1 in the supplemental material). During the 4-year period, the number of infection-related isolates was found to increase more slowly than the number of screening or of unknown source isolates. A nearly twofold decrease in the proportion of clinical versus total isolates was observed between 2013 and 2014 (Fig. 1B). Of the 172 isolates associated with disease, 122 were isolated from urine (71%), 15 from blood samples, 8 from deep site samples, 8 from wound samples, 9 from the respiratory tract, 3 from vaginal samples, 3 from pus, 3 from bile, and 1 from the skin of a newborn. All these isolates were previously identified as carbapenemase producers by PCR.

### Diversity of CP-*Ec* isolates as assessed by whole-genome sequencing.

The genome sequences of the 713 CP-*Ec* isolates were first analyzed following *de novo* assembly. For each isolate, we determined its MLST type (Achtman scheme), phylogroup (ClermonTyping), and antibiotic resistance gene (ARG) content. We also searched for mutations in the QRDRs of *gyrA*, *parC*, and *parE* and for mutations in *ftsI*, *ompC*, and *ompF* we previously identified as associated with CP-*Ec* disseminated lineages (10) (Table S1). F-NRC CP-*Ec* bacteria were assigned to 168 different STs, including six new allelic combinations. ST38 was the most prevalent ST (*n* = 92, 12.9%), followed by ST10 (*n* = 67, 9.4%), ST410 (*n* = 64, 9%), and ST167 (*n* = 34, 4.8%). Ten additional STs were represented by at least 10 isolates (see Fig. S1A and Table S1 in the supplemental material), and 154 STs were represented by less than 10 isolates, including 102 STs with a single isolate.

10.1128/mSystems.01169-21.1 FIG S1 Per-year analysis of the origin of the isolates received by the F-NRC. (A) Proportions of the isolates belonging to the different STs. The total number of isolates is indicated within parentheses. The large proportion of ST90 isolates collected in 2012 is linked to a local outbreak. (B) Proportions of the isolates collected in screening and infection situations as a function of the year and phylogroup. The absolute number of isolates for each class is indicated inside the bars. Download FIG S1, PDF file, 0.02 MB. Copyright © 2022 Patiño-Navarrete et al. 2022 Patiño-Navarrete et al. https://creativecommons.org/licenses/by/4.0/ This content is distributed under the terms of the Creative Commons Attribution 4.0 International license.

**FIG 2:**
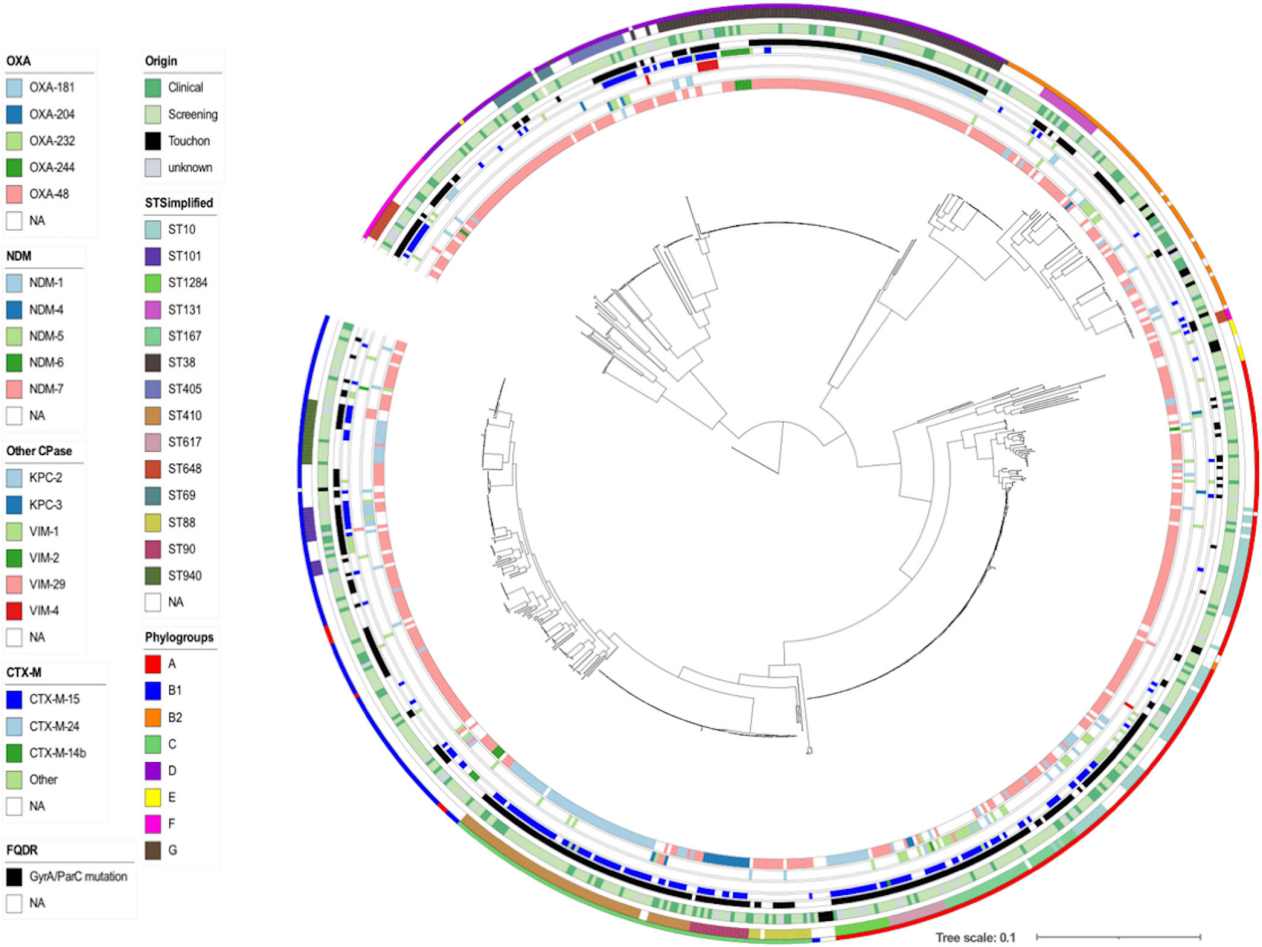
Core genome phylogeny of CP-Ec isolates received by the F-NRC. ML phylogeny of the 713 CP-*Ec* isolates was built with RAxML (45) from 372,238 core SNPs after sequence alignment on MG1655-K12 (NC_000913.3) selected as the best reference. The genome sequence of Escherichia fergusonii strain ATCC 35469 (NC_011740.1) was used as an outgroup for the phylogenetic analysis. Genomes from Touchon et al. (40) were also incorporated into the analysis. Genomic features are indicated as in the figure key (left) from the inside to the outside circles: carbapenemases of the OXA, NDM, and other types, CTX-M ESBL, mutations in *gyrA* and *parC* QRDR region (FQ resistance), origin, main ST, phylogroups. Reference genomes from Touchon et al. (40) are indicated as Touchon. NA, not available.

We next performed a core genome phylogeny following read mapping using strain E. coli MG1655 (NCBI reference sequence NC_000913.3) as the reference genome sequence. The phylogenetic tree, estimated from 372,238 core single nucleotide polymorphisms (SNPs) was consistent with the results of phylogroup determination by using *in silico* ClermonTyping (12) except for a few isolates (Fig. 2). In agreement with the MLST-based analysis, this tree showed a broad diversity of CP-*Ec* isolates belonging to the eight phylogroups and three dominant clades corresponding to CC10 (phylogroup A; including ST10, ST167, and ST617), CC23 (phylogroup C; including ST410, ST88, and ST90) and ST38 (phylogroup D) with 161, 97, and 98 isolates, respectively, accounting for 49.9% of the F-NRC CP-*Ec* isolates analyzed (Fig. 2). Phylogroup B2 isolates represented 11% of the total isolates (*n* = 80), and only 17 CP-ST131 isolates were identified. Fluctuations in the proportions of the main STs could be observed during the 4 years of the analysis, but no clear tendency could be identified (Fig. S1A).

Infection-related and screening isolates were intermixed throughout the phylogeny (Fig. 2). However, an enrichment in infection-related isolates was observed in phylogroup C (Pearson’s chi-squared test, *P* < 0.02, degrees of freedom [df] = 1) and phylogroup B2 (Pearson’s chi-squared test, *P* < 0.0005, df = 1) (Fig. S1B). In phylogroup B2, 5 out of 9 ST127 isolates, 5 out of 17 ST131 isolates, and 7 out of 8 ST636 isolates were responsible for UTIs (Table S1).

### Diversity of antibiotic resistance genes carried by CP-*Ec* isolates.

**FIG 3:**
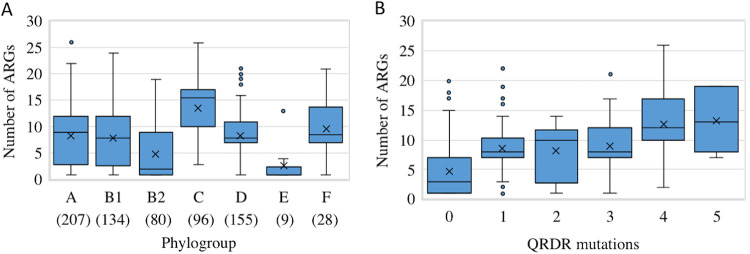
Resistance gene content of the F-NRC CP-*Ec* isolates. (A) Box plot representation of the ARG content according to the phylogroup. The number of isolates belonging to each phylogroup is indicated within parentheses. The limits of the box indicate the lower and upper quartile. Outliers are indicated by points above the maximum value. (B) Relationships between the number of ARGs and the number of mutations in QRDRs. In the box plot representations, the median is indicated by an horizontal bar, and the mean is indicated by a × symbol.

The number of acquired ARGs was found to vary from 1 to 26 among the 713 CP-*Ec* isolates. The median was higher in phylogroup C isolates (median, 16) and lower in isolates from phylogroup B2 (median, 2) compared to other phylogroups (medians for phylogroup A, B1, and D were 9, 8, and 8, respectively) (Fig. 3A). An ESBL of the CTX-M family was present in 40.7% (*n* = 290) of the isolates, with a predominance of *bla*_CTX-M-15_ gene (*n* = 205) (Table S1). Mutations in g*yrA*, *parC* and/or *parE* potentially leading to fluoroquinolone (FQ) resistance occurred in 425 CP-*Ec* isolates (59.6%), with mutations in *gyrA*, *parC*, and *parE* identified in 412, 309, and 261 isolates, respectively (Table S1). Up to five mutations in QRDRs were identified in 19 isolates, and 250 isolates had four mutations in QRDRs, suggesting they have been subjected to long-term evolution under antibiotic pressure, including FQ (Table S1). Globally, a higher number of mutations in QRDRs was associated with a higher number of resistance genes (Fig. 3B), furthering the link between the number of QRDR mutations and a likely evolution under antibiotic selective pressure for CP-*Ec* isolates.

Four isolates from 2015, two ST648 isolates collected in the same hospital at 1-week interval, one ST216 isolate and one ST744 isolate, carried a *mcr-9* gene conferring resistance to colistin (Table S1). Although not associated with infection, the four isolates were MDR, carrying 13 to 20 acquired ARGs in addition to *mcr-9* and carbapenemase genes (*bla*_VIM-1_ for three of the isolates, *bla*_NDM-1_ and *bla*_OXA-48_ for one isolate). Determination of the MIC for colistin for these four isolates were at the resistance breakpoint (MIC = 2 mg/L).

### Diversity of carbapenemase genes.

Carbapenemase genes identified by WGS (Table S1) were in agreement with molecular data collected by the F-NRC laboratory. The *bla*_OXA-48_ and *bla*_OXA-181_ genes were the most frequent carbapenemase genes detected in 464 (65%) and 101 (14.1%) E. coli isolates, respectively. Twenty-nine other isolates carried minor *bla*_OXA-48-like_ genes (Table S1). *bla*_NDM_ family genes were detected in 14.9% (106/713) of the CP-*Ec* isolates (*bla*_NDM-5_ in 49 isolates and *bla*_NDM-1_ in 41 isolates). Three additional *bla*_NDM_ alleles were identified: *bla*_NDM-7_ (*n* = 10) including a variant coding for a NDM7-like carbapenemase with a S24G mutation, *bla*_NDM-4_ (*n* = 5), and *bla*_NDM-6_ (*n* = 1). Four different *bla*_VIM_ alleles were detected in 18 isolates: *bla*_VIM-1_ (*n* = 8), *bla*_VIM-4_ (*n* = 8), *bla*_VIM-2_ (*n* = 1), and *bla*_VIM-29_ (*n* = 1). Only five E. coli isolates expressed *bla*_KPC-2_ (*n* = 2) or *bla*_KPC-3_ (*n* = 3) alleles. In 12 isolates, two different carbapenemase genes were found; *bla*_OXA-48_/*bla*_NDM-1_ was the most frequent cooccurrence (*n* = 5) (Table S1).

**FIG 4:**
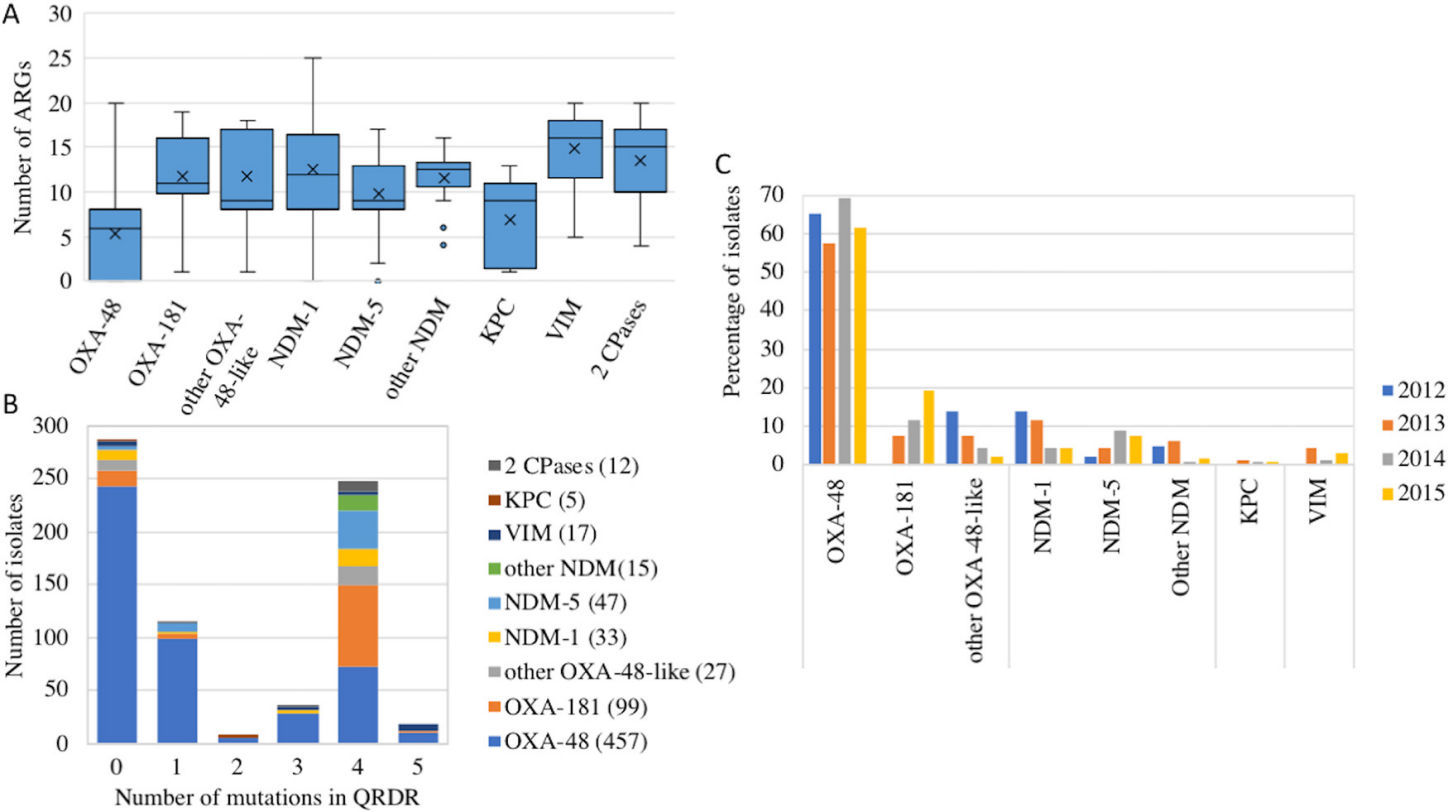
Carbapenemase gene content of the F-NRC CP-*Ec* isolates. (A) Relationship between the carbapenemase allele and the ARG content. In the box plot representation, the median is indicated by an horizontal bar and the mean by a × symbol. The limits of the box indicate the lower and upper quartile. Outliers are indicated by points above the maximum value. (B) Number of isolates carrying a specific carbapenemase allele according to the number of QRDR mutations in their genomes. (C) Per-year evolution of the percentage of isolates carrying the different carbapenemase alleles in the 2012–2015 period. The year of isolation is indicated as in the figure key (right). Given their small number (*n* = 22), strains isolated before 2012 are not indicated.

The analysis of the number of additional ARGs and of mutations in QRDR regions showed that, compared with other carbapenemase genes, the presence of *bla*_OXA-48_ was frequently associated with less resistant isolates (Fig. 4A and B). In particular, it was the only ARG in 117 isolates of the 457 (24.5%) encoding this carbapenemase, including 23 infection-related isolates. Of those isolates, only five showed mutations in QRDRs. Conversely, only two *bla*_NDM-1_ gene-carrying isolates of 42 isolates and one *bla*_NDM-5_ gene-carrying isolates of 49 isolates carried no other ARG.

A temporal analysis of the proportion of isolates displaying *bla*_OXA-48_ did not reveal significant variations (Pearson’s chi-squared test, *P* = 0.05, df = 3) during the 2012–2015 period (Fig. 4C). In contrast, the frequency of *bla*_OXA-181_ relative to other alleles was found to significantly increase from 0% in 2012 to 18.9% in 2015 (Pearson’s chi-squared test, *P* < 0.0002, df = 3) (Fig. 4C).

**FIG 5:**
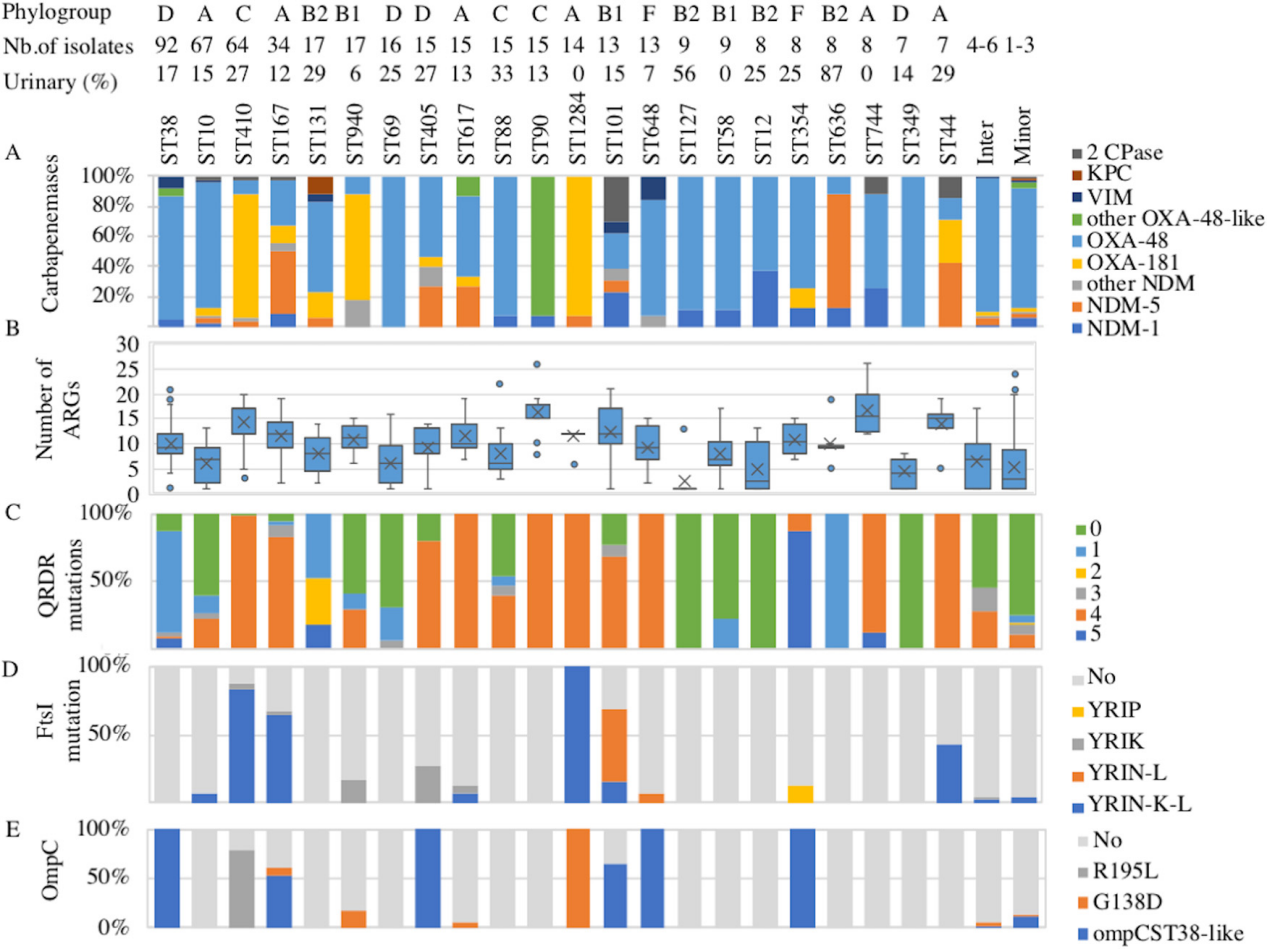
Features of the main STs associated with CP-*Ec* isolates in France. At the top of the figure, for each ST, the phylogroup, the total number of isolates, and the number of isolates associated with urinary tract infections are indicated. Intermediate (Inter) and minor ST indicate ST represented by four to six isolates and one to three isolates, respectively. (A) Proportion of the isolates according to the carbapenemase allele in each ST. (B) Box plot representation of the ARGs content according to the ST. (C) Proportion of isolates according to the number of mutations in QRDR. (D) Proportion of isolates with a specific PBP3 allele. PBP3 characterized by a YRIP, YRIK, or YRIN insertion between positions 333 and 334, the YRIN insertion is generally associated with a A413V mutation (YRIN-L) and less frequently also with a E349K mutation (YRIN-K-L) (10). (E) Proportion of isolates carrying specific mutations in *ompC*: R195L, G138D or acquisition of an ST38-like *ompC* allele by recombination (10).

We observed some association between the carbapenemase gene and the ST. Of the 22 STs with at least seven F-NRC CP-*Ec* isolates, 14 predominantly displayed *bla*_OXA-48_ (Fig. 5A); *bla*_OXA-48_ was also predominant in STs represented by six or less isolates. In three lineages, ST410, ST940, and ST1284, *bla*_OXA-181_ was the predominant allele. These three STs grouped 74% of the *bla*_OXA-181_ gene carrying E. coli isolates from the F-NRC, and ST410 accounted for 50.5% of them. Finally, *bla*_OXA-204_ was the most prevalent allele in ST90, and *bla*_NDM-5_ was the predominant allele in ST636.

### Characteristics of the STs preferentially associated with F-NRC CP-*Ec* isolates.

Eight of the 14 STs with more than 10 CP-*Ec* isolates (ST410, ST167, ST940, ST405, ST617, ST90, ST1284, and ST101) were characterized by a larger number of ARGs (median ≥ 10) and a larger number of mutations in QRDRs compared to ST38, ST10, ST131, ST69, ST88, and ST represented by less than seven isolates (Fig. 5B and C). ST648 isolates have an intermediate position, being highly mutated in QRDRs but less rich in ARGs. Analysis of CP-*Ec* isolates from the F-NRC for polymorphisms in *ftsI* revealed that 131 (18.4%) had a 4-amino-acid (aa) insertion between positions 333 and 334 of PBP3, the insertion being particularly prevalent in ST101, ST167, ST410, and ST1284 (Table S1 and Fig. 5D). Similarly, *ompC* alleles we previously characterized as modifying susceptibility to antibiotics were more prevalent in some STs: an ST38-like *ompC* allele resulting from a recombination event or a G138D mutation was present in 33 and 26 isolates belonging to six different STs, whereas the R195L mutation was observed in 50 isolates all from ST410 (Table S1 and Fig. 5E). In addition, 144 isolates belonging to phylogroups D and F possessed the ST38-like allele inherited vertically. On the other hand, the *ompF* gene was pseudogeneized in 47 isolates (Table S1).

To analyze the phylogenetic relationships between F-NRC Cp-*Ec* and other Cp-*Ec* isolates collected worldwide, we built ST-based maximum likelihood trees for the 14 STs with more than 10 isolates and included to this analysis genome sequences publicly available (Table S2). Together with the analysis of QRDR mutations and mutations in *ftsI* and *ompC*, this showed that 51 (80%) of the ST410 (Fig. S2A), 17 (49%) of the ST167 (Fig. S2B), 3 (33%) of the ST405 (Fig. S2C), and 8 (62%) of the ST101 (Fig. S2D) F-NRC CP-*Ec* isolates belonged to internationally disseminating MDR subclades enriched in CP we previously identified (10). Subclades characterized by mutations in *ftsI* and *ompC* mainly expressed *bla*_OXA-181_ (ST410) or *bla*_NDM_ (other lineages). Other isolates belonging to these STs were dispersed on their respective phylogenies and with no sign of clonal dissemination for most of them.

10.1128/mSystems.01169-21.2 FIG S2 Core genome phylogenies of the main STs characterized by clades disseminating internationally. (A) ST410; (B) ST167; (C) ST405; (D) ST101. Phylogenies were based on genome sequences of 64 CP-*Ec* isolates from the F-NRC and 146 genome sequences from the NCBI database (A), 35 CP-*Ec* isolates from the F-NRC and 134 genome sequences from the NCBI database (B), 15 CP-*Ec* isolates from the F-NRC and 145 genome sequences from the NCBI database (C), and 13 CP-*Ec* isolates from the F-NRC and 194 genome sequences from the NCBI database (D). Core genome (ST410, 3,522,000 nt; ST167, 3,276,000 nt; ST405, 3,685,000 nt; ST101, 3,774,000 nt) alignments of the *de novo* assemblies on the sequences of GCA_001442495.1 (ST410), GCA_003028815.1 (ST167), GCA_002142675.1 (ST405), and GCA_002163655.1 (ST101) used as reference were performed by using Parsnp (T. J. Treangen, B. D. Ondov, S. Koren, and A. M. Phillippy, Genome Biol 15:524, 2014, https://doi.org/10.1186/preaccept-2573980311437212); ML phylogeny was built with RAxML (A. Stamatakis, Bioinformatics 30:1312−1313, 2014, https://doi.org/10.1093/bioinformatics/btu033) from 6,176 (ST410), 10,393 (ST167), 37,482 (ST405), and 19,967 (ST101) core SNPs after removing recombined regions with gubbins (N. J. Croucher, A. J. Page, T. R. Connor, A. J. Delaney, et al., Nucleic Acids Res 43:e15, 2015, https://doi.org/10.1093/nar/gku1196). The genome sequences of CNR93E7 (ST88), CNR93D10 (ST746), CNR73I9 (ST115), and CNR93I2 (ST906) were used as outgroups for the phylogenetic analyses of ST410, ST167, ST405, and ST101, respectively. The origin from the F-NRC is indicated by red triangles close to the isolate name. Other genomic features are indicated as indicated in the figure key (left) from the inside to the outside lines: carbapenemases of the OXA, NDM, and other types; number of mutations in *gyrA* and *parC* QRDR region (FQ resistance), number of ARGs, mutations in *ftsI*, *ompC*, *ompF*; and geographical origin. Download FIG S2, PDF file, 0.9 MB. Copyright © 2022 Patiño-Navarrete et al. 2022 Patiño-Navarrete et al. https://creativecommons.org/licenses/by/4.0/ This content is distributed under the terms of the Creative Commons Attribution 4.0 International license.

**FIG 6:**
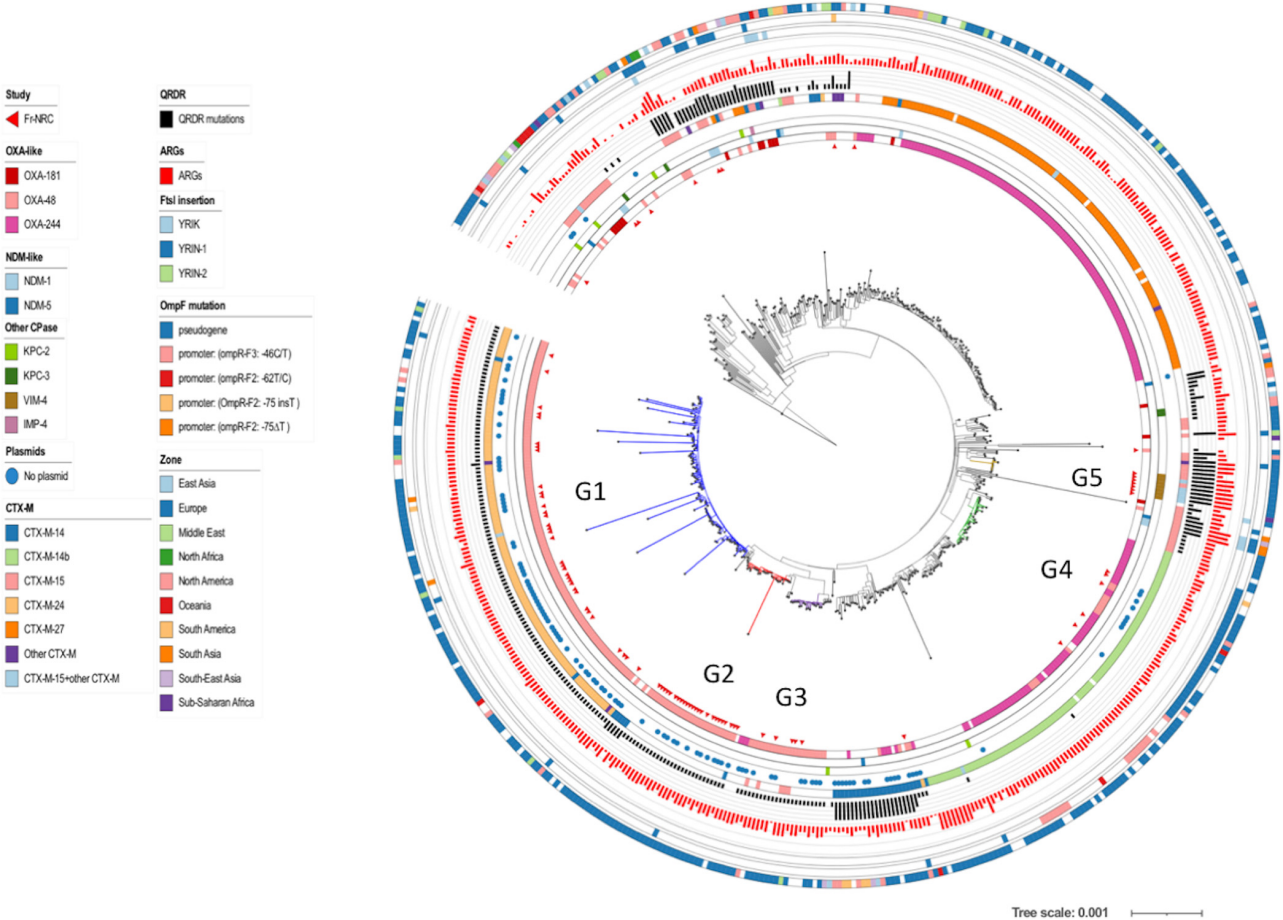
Core genome phylogeny of ST38 isolates. ML Phylogeny was based on genome sequences of 92 CP-*Ec* from the F-NRC and 464 genome sequences retrieved from EnteroBase and from the NCBI database, including 331 carrying a carbapenemase gene. A core genome (2,900,000-nt) alignment of the *de novo* assemblies on the sequence of GCA_005886035.1 used as a reference was performed by using Parsnp (47); ML phylogeny was built with RAxML (45) from 6,170 core SNPs after removing recombined regions with Gubbins (48). The genome sequence of CNRC6O47 (ST963) was used as an outgroup for the phylogenetic analysis. F-NRC isolates are indicated by red triangles (inner circle). Other genomic features are indicated as indicated in the figure key (left) from the inside to the outside circles: carbapenemases of the OXA, NDM, and other types; absence of any plasmid of any Inc type as identified by using PlasmidFinder (14); ESBL of the CTX-M types; number of mutations in *gyrA* and *parC* QRDRs (FQ resistance); number of ARGs; mutations in *ftsI*, *ompC*, and *ompF*; and geographical origin. The four OXA-48-like clades (G1, G2, G3, and G4) clustering most French isolates are colored in blue, red, violet, and green. A fifth clade (G5) corresponding to a possible outbreak of VIM-4 isolates in east of Paris is colored in brown.

**FIG 7:**
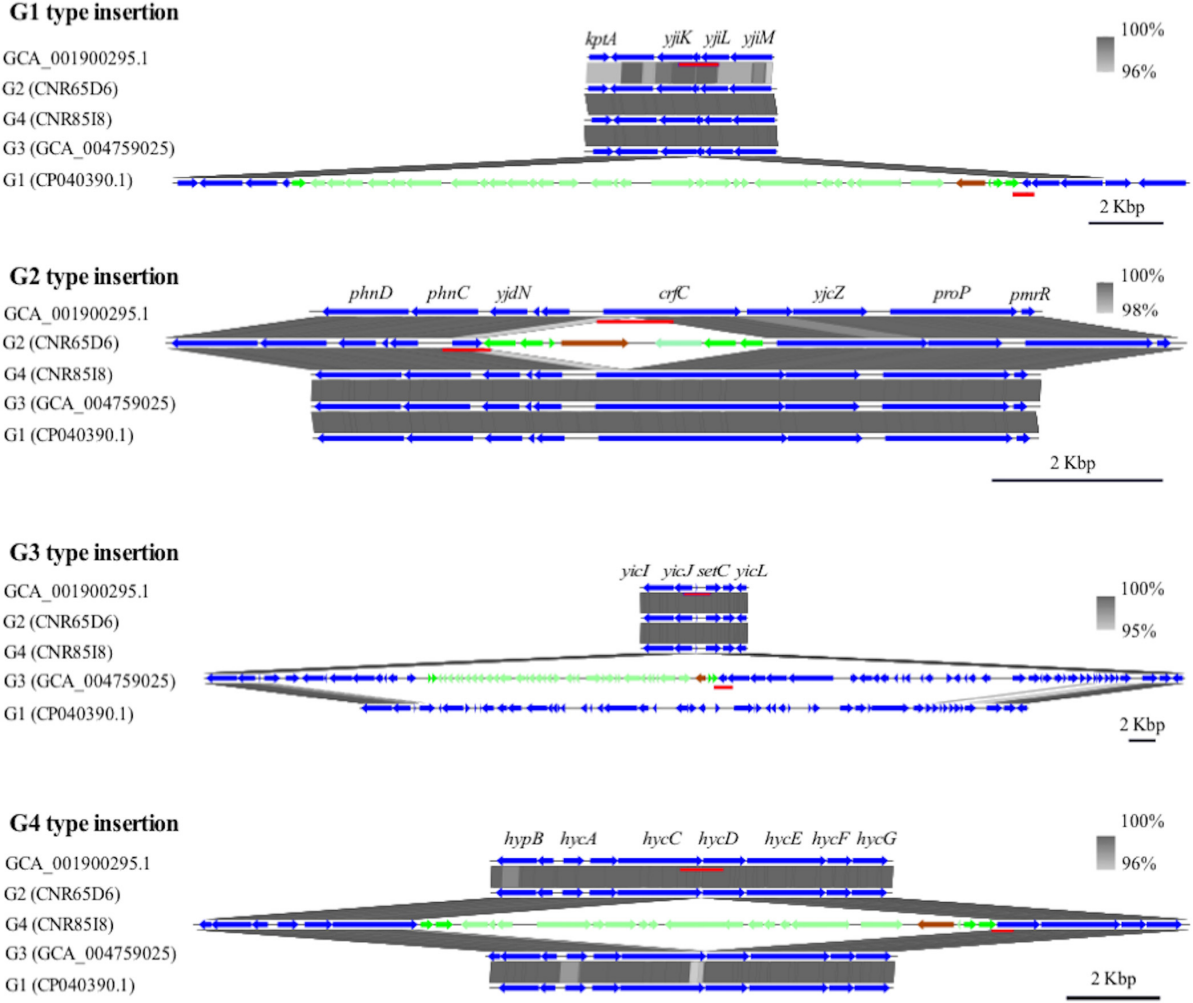
*bla*_OXA-48_ integration sites of ST38 CP-*Ec* clades 1, 2, 3, and 4. The genome sequences of GCA_005886035.1 (CP040390.1), CNR65D6, GCA_004759025.1 (CP038505.1), and CNR85I8 were chosen as representative genomes of clusters 1, 2, 3, and 4, respectively, and aligned, by using BlastN, against the sequence of GCA_001900295.1 (CP010116.1), an ST38 isolate that does not carry a carbapenemase gene and whose genome sequence was complete. Gene annotations were derived from strain MG1655 (NC_000913.3). The regions surrounding the *bla*_OXA-48_ insertion sites in each clade were more precisely aligned against the corresponding sequences of CP010116.1 and of representative genomes of the other clades by using Easyfig (52). *bla*_OXA-48_ is represented by a brown arrow, cointegrated genes of pOXA-48 origin are indicated by light green arrows, and the IS*1* transposase is indicated by a green arrow. Sequences used to perform the BLAST analysis on Illumina contigs are underlined in red. It is interesting to note that, in the G3 clade, *bla*_OXA-48_ integration occurs in a genomic island inserted at tRNASec. Another genomic island is present in G1-type strains but does not carry *bla*_OXA-48_.

ST38 corresponded to the most represented ST (*n* = 92) among the studied CP-*Ec*. The phylogenetic reconstruction together with 314 additional nonredundant CP-*Ec* genome sequences retrieved from EnteroBase (http://enterobase.warwick.ac.uk/) and 150 non-CP isolates from NCBI provided evidence that 80.4% (*n* = 67) of the French isolates clustered into four clades (Fig. 6). Three of the clades, G1 (*n* = 35), G2 (*n* = 25), and G4 (*n* = 4) only contained isolates encoding *bla*_OXA-48_, while G3 (*n* = 7) included isolates expressing *bla*_OXA-48_ or its single nucleotide derivative *bla*_OXA-244_. All but one isolate in G1 expressed *bla*_CTX-M-24_, while isolates of G3 expressed *bla*_CTX-M-14b_. The phylogenetic analysis provided evidence of worldwide dissemination of G1, G3, and G4 clades and multiple introduction in France. Strikingly, among the 28 isolates of the G2 clade, 24 were collected in France and four in the Netherlands, suggesting at this time a more regional dissemination. In none of the isolates of the four clades could an IncL plasmid, that generally carries *bla*_OXA-48_ (13), be identified by PlasmidFinder (14). It suggests a chromosomal integration as previously shown among ST38 isolates collected in the United Kingdom (15). Analysis of representative isolates of the G1 and G3 cluster (G1, GCA_005886035.1; G3, GCA_004759025.1), whose genome sequences were completely assembled, confirmed that *bla*_OXA-48_ and *bla*_OXA-244_, in these two isolates, respectively, were chromosomally integrated. To assess the genetic support of *bla*_OXA-48_ in the two other clades, we have determined the complete genome sequence of two isolates belonging to G2 (CNR65D6) and G4 (G4:CNR85I8) by combining long-read Pacbio and Illumina sequencing and found that both possess a chromosomally inserted *bla*_OXA-48_ gene. pOXA-48 plasmid sequences of various lengths were cointegrated with *bla*_OXA-48,_ the whole sequence being surrounded by IS*1* insertion sequences with different insertion sites for the four clades (Fig. 7). BlastN analyses on the assembled Illumina sequences of other isolates of each clade showed that isolates of the same clade share the same insertion sites (see Materials and Methods). A fifth clade, composed of seven closely related French isolates corresponded to a possible outbreak east of Paris, France, between December 2014 and December 2015. These isolates are predicted to be highly resistant, as they are carrying in addition to *bla*_VIM-4,_ *bla*_CTX-M-15_, as well as 15 to 18 additional ARGs and five mutations in QRDRs.

ST10 E. coli isolates are commensals of a variety of mammals and bird species (16), and studies in different contexts have shown that this ST is also associated with ARG carriage (17, 18). The 67 F-NRC ST10 CP-*Ec* isolates were distributed throughout the ST10 phylogeny. Nonetheless, 31 isolates belonged to a clade enriched with carbapenemase producers (Fig. S3A, in green) that mainly expressed *bla*_OXA-48_, with the exception of three isolates expressing *bla*_OXA-181_, three expressing *bla*_NDM-5_, and one expressing *bla*_VIM-4_. Two of the *bla*_OXA-181_-expressing isolates and the three *bla*_NDM-5_-expressing isolates were closely related, shared a CTX-M-27 ESBL, four mutations in QRDRs, and a *ftsI* allele with a 4-aa YRIN insertion (Fig. S3A, in red).

10.1128/mSystems.01169-21.3 FIG S3 Core genome phylogenies of ST10 and ST131 isolates. (A) ST10 phylogeny based on the genome sequences of 67 CP-*Ec* isolates from the F-NRC and 153 genome sequences from the NCBI. (B) ST131 phylogeny based on the genome sequences of 17 CP-*Ec* isolates from the F-NRC and 462 genome sequences from the NCBI. Core genome (ST10, 813,000 nt; ST131, 1,641,000 nt) alignments of the *de novo* assemblies on the sequence of MG1655 (ST10) or GCA_000285655.3 (ST131) used as references were performed by using Parsnp (T. J. Treangen, B. D. Ondov, S. Koren, and A. M. Phillippy, Genome Biol 15:524, 2014, https://doi.org/10.1186/preaccept-2573980311437212); ML phylogeny was built with RAxML (A. Stamatakis, Bioinformatics 30:1312−1313, 2014, https://doi.org/10.1093/bioinformatics/btu033) from 20,245 (ST10) and 14,366 (ST131) core SNPs after removing recombined regions with Gubbins (N. J. Croucher, A. J. Page, T. R. Connor, A. J. Delaney, et al., Nucleic Acids Res 43:e15, 2015, https://doi.org/10.1093/nar/gku1196). The genome sequences of CNR93D10 (ST746) and CNRAL47G10 (ST640) were used as outgroups for the phylogenetic analyses of ST10 and ST131, respectively. F-NRC isolates are indicated by red triangles (inner circle). Other genomic features are indicated as indicated in the figure key (left) from the inside to the outside circles: carbapenemases of the OXA, NDM, and other types; number of mutations in *gyrA* and *parC* QRDR region (FQ resistance), number of ARGs, mutations in *ftsI*, *ompC*, and *ompF*; and geographical origin. The ST10 clade enriched in CP-*Ec* is indicated in green and the subclade with the YRIN insertion in PBP3 is indicated in red. Note that the circle for *ftsI* mutations is absent in ST131, as no YRIN-like duplication in PBP3 was identified among the analyzed isolates. Download FIG S3, PDF file, 1.3 MB. Copyright © 2022 Patiño-Navarrete et al. 2022 Patiño-Navarrete et al. https://creativecommons.org/licenses/by/4.0/ This content is distributed under the terms of the Creative Commons Attribution 4.0 International license.

ST131 isolates are major contributors for extraintestinal infections and the main factor responsible for the dissemination of CTX-M-15 ESBL (19, 20). ST131 evolution has been thoroughly analyzed, and four main lineages A, B, C1, and C2 have been described (21). We identified only 17 ST131 CP-*Ec* isolates received by the F-NRC (Fig. 2), 10 belonged to lineage A, while the others were scattered in the three other lineages (Fig. S3B). The main carbapenemase associated with these isolates was OXA-48 (*n* = 10). We also did not observe any association with the carriage of *bla*_CTX-M_ genes, as only four ST131 CP-*Ec* isolates carried a *bla*_CTX-M-15_ gene. These results are in accordance with the United Kingdom survey (8) and our previous observations from sequences retrieved from public databases (10).

ST940 (phylogroup B1) and ST1284 (phylogroup A) represented 2.4% (*n* = 17) and 1.9% (*n* = 14) of the sequenced isolates, while they were poorly represented in sequence databases. For instance, in May 2021, only 83 and 103 genome sequences could be retrieved from EnteroBase for these STs, respectively, but with 43% (*n* = 36) and 22% (*n* = 22) carrying a carbapenemase gene, respectively. This suggested that ST940 and ST1284 were associated with the dissemination of carbapenemase genes. Phylogenetic trees were drawn by adding the nonredundant sequences from EnteroBase to those of the F-NRC and NCBI. For ST940 (Fig. S4A), this revealed a well-differentiated subclade of 18 isolates from Asia, Australia, and Europe carrrying *bla*_NDM-5_ and sharing mutations in *ftsI* and *ompC*. Isolates from the F-NRC were distributed on the tree, showing that the overrepresentation of ST940 in France did not result from local outbreaks. Three F-NRC isolates belonged to a second smaller subclade of *bla*_NDM_-expressing isolates, also mutated in *ftsI* and *ompC*. In contrast, in ST1284, 13 isolates carrying *bla*_OXA-181_ were found to be closely related (Fig. S4B). Metadata analysis showed that 12 of them were recovered from the same health facility in the Paris suburb during a 1-month period in 2015, demonstrating a local outbreak origin. A fourteenth unrelated isolate expressed *bla*_NDM-5_. The F-NRC isolates belonged to a clade characterized by four mutations in QRDR determinants, a YRIN duplication in PBP3, a G137D mutation in OmpC and containing additional OXA-181- or NDM-producing isolates.

10.1128/mSystems.01169-21.4 FIG S4 Core genome phylogeny of ST940 and ST1284 isolates. (A) ST940 phylogeny based on genome sequences of 17 CP-*Ec* isolates from the F-NRC and 88 genome sequences from EnteroBase and the NCBI database. (B) ST1284 phylogeny based on genome sequences of 14 CP-*Ec* isolates from the F-NRC and 60 genome sequences from EnteroBase and the NCBI database. A core genome (ST940, 3,639,000 nt; ST1284, 3,779,000 nt) alignment of the *de novo* assemblies on the sequence of ST940: ESC_LB2149 and ST1284: ESC_LB2152AA (from EnteroBase) used as reference was performed by using Parsnp (T. J. Treangen, B. D. Ondov, S. Koren, and A. M. Phillippy, Genome Biol 15:524, 2014, https://doi.org/10.1186/preaccept-2573980311437212); ML phylogeny was built with RAxML (A. Stamatakis, Bioinformatics 30:1312−1313, 2014, https://doi.org/10.1093/bioinformatics/btu033) from 13,859 (ST940) and 2,009 (ST1284) core SNPs after removing recombined regions with Gubbins (N. J. Croucher, A. J. Page, T. R. Connor, A. J. Delaney, et al., Nucleic Acids Res 43:e15, 2015, https://doi.org/10.1093/nar/gku1196). The genome sequences of CNR98G1 (ST3022) and MG1655 (ST10) were used as outgroups for the phylogenetic analyses of ST940 and ST1284, respectively. F-NRC isolates are indicated by red triangles in thefirst column on the left. Other genomic features are as indicated in the figure key (left) from the left to the right columns: carbapenemases of the OXA, NDM, and other types; number of mutations in *gyrA* and *parC* QRDR region (FQ resistance); number of ARGs; mutations in *ftsI*, *ompC*, and *ompF*; and geographical origin. Download FIG S4, PDF file, 0.3 MB. Copyright © 2022 Patiño-Navarrete et al. 2022 Patiño-Navarrete et al. https://creativecommons.org/licenses/by/4.0/ This content is distributed under the terms of the Creative Commons Attribution 4.0 International license.

Of the 15 ST90 CP-*Ec* isolates, 9 encoding the *bla*_OXA-204_ allele were isolated in hospitals east of Paris between August 2012 to April 2013 and suspected to be associated with the use of a contaminated endoscope (22). Isolates closely related to this outbreak reappeared in 2014 (three times) and in 2015 (twice), also in hospitals located east of Paris. They belonged to a MDR clade characterized by four mutations in QRDRs (Fig. S5A). Finally, in the four last STs with more than 10 CP-*Ec* isolates (ST69, ST88, ST617, and ST648), French CP-*Ec* isolates were distributed throughout their respective phylogenetic trees (Fig. S5B and S5C and Fig. S6A and S6B) with no sign of clonal dissemination, except for a small cluster of five ST69 isolates from two geographical regions and expressing *bla*_OXA-48_.

10.1128/mSystems.01169-21.5 FIG S5 Core genome phylogenies of ST69, ST88, and ST90 isolates. (A) ST90 phylogeny based on the 15 CP-*Ec* isolates from the F-NRC and 38 genome sequences from the NCBI database. (B) ST69 phylogeny based on the 16 CP-*Ec* isolates from the F-NRC and 244 genome sequences from the NCBI database. (C) ST88 phylogeny based on the 15 CP-*Ec* isolates from the F-NRC and 99 genome sequences from the NCBI database. Core genome (ST69, 2,735,000 nt; ST88, 3,472,000 nt; ST90, 3,801,000 nt) alignments of the *de novo* assemblies on the sequence of GCA_002443135.1 (ST69), GCA_002812685.1 (ST88), or GCA_001900635.1 (ST90) used as reference were performed by using Parsnp (T. J. Treangen, B. D. Ondov, S. Koren, and A. M. Phillippy, Genome Biol 15:524, 2014, https://doi.org/10.1186/preaccept-2573980311437212); ML phylogeny was built with RAxML (A. Stamatakis, Bioinformatics 30:1312−1313, 2014, https://doi.org/10.1093/bioinformatics/btu033) from 4,596 (ST69), 12,557 (ST88), or 2,878 (ST90) core SNPs after removing recombined regions with Gubbins (N. J. Croucher, A. J. Page, T. R. Connor, A. J. Delaney, et al., Nucleic Acids Res 43:e15, 2015, https://doi.org/10.1093/nar/gku1196). The genome sequences of CNR33D9 (ST394), CNR83B9 (ST410), and CNR88B9 (ST847) were used as outgroups for the phylogenetic analyses of ST69, ST88, and ST90, respectively. F-NRC isolates are indicated by red triangles (first column on the left). Other genomic features are indicated as indicated in the figure key (left) from the left to the right columns: carbapenemases of the OXA, NDM, and other types; number of mutations in *gyrA* and *parC* QRDR region (FQ resistance); number of ARGs; mutations in *ftsI*, *ompC*, and *ompF*; and geographical origin. Download FIG S5, PDF file, 0.4 MB. Copyright © 2022 Patiño-Navarrete et al. 2022 Patiño-Navarrete et al. https://creativecommons.org/licenses/by/4.0/ This content is distributed under the terms of the Creative Commons Attribution 4.0 International license.

10.1128/mSystems.01169-21.6 FIG S6 Core genome phylogeny of ST617 and ST648 isolates based on genome sequences of ST617 genome sequences of 15 CP-*Ec* isolates from the F-NRC and 65 genome sequences from the NCBI database (A) and ST648genome sequences of 14 CP-*Ec* isolates from the F-NRC and 126 genome sequences from the NCBI database (B). A core genome (ST617, 3,407,000; ST648, 3,501,000 nt) alignment of the *de novo* assemblies on the sequences of GCA_002142695.1 (ST617) or GCA_004138645.1 (ST648) used as reference was performed by using Parsnp (T. J. Treangen, B. D. Ondov, S. Koren, and A. M. Phillippy, Genome Biol 15:524, 2014, https://doi.org/10.1186/preaccept-2573980311437212); ML phylogeny was built with RAxML (A. Stamatakis, Bioinformatics 30:1312−1313, 2014, https://doi.org/10.1093/bioinformatics/btu033) from 15,030 (ST617) or 3,782 (ST648) core SNPs after removing recombined regions with Gubbins (N. J. Croucher, A. J. Page, T. R. Connor, A. J. Delaney, et al., Nucleic Acids Res 43:e15, 2015, https://doi.org/10.1093/nar/gku1196). The genome sequences of CNR93D10 (ST746) and CNR71A8 (ST1485) were used as outgroups for the phylogenetic analyses of ST617 and ST648, respectively. F-NRC isolates are indicated by red triangles close to the isolate name. Other genomic features are indicated as indicated in the figure key (left) from the left to the right columns: carbapenemases of the OXA, NDM, and other types; number of mutations in *gyrA* and *parC* QRDR region (FQ resistance); number of ARGs; mutations in *ftsI*, *ompC*, and *ompF*; and geographical origin. Download FIG S6, PDF file, 0.4 MB. Copyright © 2022 Patiño-Navarrete et al. 2022 Patiño-Navarrete et al. https://creativecommons.org/licenses/by/4.0/ This content is distributed under the terms of the Creative Commons Attribution 4.0 International license.

In addition to potential outbreaks detected in ST38 (*bla*_VIM-4_), ST90 (*bla*_OXA-204_), and ST1284 (*bla*_OXA-181_), we also found evidence for another potential outbreak among isolates belonging to ST359. The five isolates carried *bla*_OXA-48_ and *bla*_CTX-M-32_ and had three mutations in QRDRs. They were isolated during January 2014, four of them in the southeastern part of France and one in the Parisian suburbs.

## DISCUSSION

The prevalence of CP-*Ec* is increasing worldwide. However, whether this reveals conjugation event in E. coli isolates by circulating CP-encoding plasmids or the emergence and dissemination of at-risk clones is still largely unknown. Here, we have analyzed, by using WGS, 691 (87.8%) of the CP-*Ec* isolates received by the F-NRC during the2012–2015 period and 22 isolates from the Bicêtre hospital strain collection (2001 to 2011) to characterize both the diversity of carbapenemase genes and of CP-*Ec* isolates in France. Altogether, 713 CP-*Ec* isolates were sequenced, representing to our knowledge the most extended collection of CP-*Ec* isolates sequenced published to date.

The number of CP-*Ec* isolates sent to the F-NRC was found to strongly increase during the 4-year period of analysis, which might be a consequence of an increased circulation of CP-*Ec* in France but also of an increased screening of potential CPE carriers at their admission to the hospital. Indeed, while the number of infection-related CP-*Ec* isolates was regularly increasing, their proportion compared to the total number of received and sequenced isolates decreased by twofold, with a clear change observed between 2013 and 2014 (Fig. 1). This is likely a consequence of the implementation of the recommendations on MDR screening of the French Public Health Advisory Board 2013 (23). However, as CP-*Ec* isolates were sent on a voluntary basis by clinical laboratories, we cannot exclude some sampling bias.

The most predominant carbapenemase allele was *bla*_OXA-48_ (65%), followed by *bla*_OXA-181_ (14.1%) which detection constantly increased from no cases in 2012 to 73 in 2015. Next was *bla*_NDM-5_ that was found to progressively substitute to the *bla*_NDM-1_ gene in the frequency of detection (Fig. 4). The predominance of *bla*_OXA-48-like_ genes and particularly of *bla*_OXA-48_, among CP-*Ec* was also noted in other studies (9, 24, 25) although in lower proportions. The small number of *bla*_KPC_ genes detected in these studies is in contrast with the situation reported in Klebsiella pneumoniae for many European countries (26). However, *bla*_OXA-48_ was found to be the most prevalent allele in K. pneumoniae in France (2) and in the Netherlands (9). This difference might result from a lower capacity for *bla*_KPC_-carrying plasmids to conjugate to E. coli compared to pOXA-48 or to a higher fitness cost. It might also be linked to sampling differences, as different thresholds of carbapenem susceptibility might have been used to collect CRE isolates among studies. Indeed, OXA-48-like carbapenemases are generally associated with lower levels of carbapenem resistance than KPC. This analysis was performed on isolates collected between 2012 and 2015 and revealed early trends in the evolution of CP-*Ec* isolated in France. It will be essential in the characterization of the genomic evolution of CP-*Ec* in more recent years, which remains to be performed.

The number of ARGs identified in the CP-*Ec* isolates from the F-NRC varied from 1 (the carbapenemase gene only) to 26, showing that carbapenemase genes were acquired not only in MDR isolates but also in an E. coli population that can be considered “naive” relative to evolution under antibiotic selective pressure and resident of the intestinal microbiota. This was particularly true for isolates producing *bla*_OXA-48_ that were generally associated with a lower number of resistance genes than isolates producing other carbapenemases, irrespective of their clinical status (Fig. 4A). In particular, 118 out of the 121 (97.5%) isolates with no other ARG than the carbapenemase gene carried *bla*_OXA-48_. Therefore, the predominance of *bla*_OXA-48_ among the carbapenemase genes identified in the F-NRC collection might mainly rely on the higher conjugative transfer rate of pOXA-48-related plasmids compared to the rates of plasmids carrying genes encoding other carbapenemases (27). pOXA-48 has indeed been shown to rapidly conjugate among *Enterobacterales* in hospitalized patients (28). This could also explain the high frequency of *bla*_OXA-48_-carrying isolates belonging to ST10, ST69, ST88, ST127, ST12, and ST58 (Fig. 5) that carried a small number of resistance genes and QRDR mutations and among which four were previously identified among the most frequent E. coli STs characterized from fecal samples (29).

On the other hand, MDR CP-*Ec* isolates may result from the transfer of carbapenem resistance genes into isolates already selected through multiple exposures to antibiotic treatments that have already acquired multiresistance plasmids or chromosomal mutations increasing their intrinsic drug resistance, such as mutations in QRDRs, in *ftsI* and/or porin genes. Alternatively, the circulation of carbapenem-resistant lineages, such as the OXA-181-producing ST410 E. coli lineage, showing a stable association between the lineage and the resistance gene, may also occur and significantly contribute to the number of CP-*Ec* isolates collected. Discriminating between both alternatives would require a more extended comparison of plasmids carried in these lineages after long-read sequencing similarly to what been done in K. pneumoniae (30). Strikingly, the analysis of *ftsI* alleles coding for a PBP3 with the 4-amino-acid duplication revealed a contrasted situation among CP-*Ec* isolates collected in France, with the duplication in *ftsI* found in 72.3%, 77.8%, and 67% of the isolates carrying *bla*_NDM-5_ (34/47), *bla*_NDM-7_ (7/9), and *bla*_OXA-181_ (68/101) genes but in only 0.7% (4/458) of the isolates carrying *bla*_OXA-48_. We previously proposed that the fixation of mutations reducing the intrinsic susceptibility to carbapenems might have favored the efficient conjugative transfer of plasmids carrying the carbapenemase genes from other CPE species in the gut (10). Transconjugants would be selected in the context of low biliary excretion of carbapenems or other β-lactams after parenteral administration. Increasing the proportion of the donor and recipient bacteria would therefore be less necessary for plasmids with high conjugative rates, such as pOXA-48 (27). Alternatively, these mutations might also have favored plasmid maintenance by increasing the resistance level and selection of the isolates during β-lactam or carbapenem treatment. Of particular concern in France are the OXA-181-producing ST410 and the NDM (NDM-1, -5, and -7) ST167 lineages that significantly contribute to the circulation of carbapenem resistance. However, our study also reveals smaller lineages, that although less frequently encountered in international studies, are nevertheless circulating. In contrast, we did not obtain evidence for clonal dissemination of CP-*Ec* ST131 lineages derived from the B2 clade responsible for *bla*_CTX-M-15_ gene dissemination (19, 20).

Despite their high frequency among the F-NRC collection, the ST38 isolates do not fall into one of the two previous categories. Indeed, while these isolates mainly express *bla*_OXA-48_ or its single point mutant derivative *bla*_OXA-244_ (31), our phylogenetic analysis revealed that a majority of them belong to four different lineages, including one mostly associated with rapid dissemination in France. In contrast with OXA-181- and NDM-producing lineages previously described, these lineages are associated with a moderate number of ARGs and QRDR mutations. None of the ST38 isolates is mutated in *ftsI*; however, we previously demonstrated that all are characterized by an *ompC* allele that encodes a porin with a reduced permeability to certain β-lactams and has disseminated into unrelated lineages by homologous recombination (10). A common feature of the four lineages is the integration of part of the pOXA-48 plasmid carrying *bla*_OXA-48_. This could have reduced a fitness cost of this plasmid and facilitated the clonal expansion of these lineages. However, ST38 isolates were identified as a infrequent colonizer of the gastrointestinal (GI) tract (29, 32). Therefore, additional features of these CP-*Ec* ST38 lineages might have contributed to their dissemination.

In conclusion, by analyzing genome sequences of CP-*Ec* isolates collected by the F-NRC, we showed that MDR lineages, enriched in carbapenemase-producing isolates are circulating in France, and that some of them, such as ST1284 isolates, are associated with outbreaks. The results of our study also suggest that the evolutionary trajectory may depend on the carbapenemase gene, *bla*_OXA-181_ or *bla*_NDM_ genes being more frequently associated with the evolution of MDR E. coli lineages characterized by mutations in *ftsI* and *ompC*. Surveillance of these mutations may be an important parameter in controlling the circulation of MDR lineages. On the other hand, carbapenemase genes are also frequently acquired through plasmid dissemination from other bacterial species. Depending on the resistance background of the receiver E. coli, this may lead to XDR or to isolates sensitive to a broad range of antibiotics. Finally, we also observed a strong and rapid dissemination of ST38 isolates that might have been favored by a reduced susceptibility to carbapenems linked to the ST38 *ompC* allele and by the chromosomal integration of the carbapenemase gene. These results strengthen our model of different evolutionary trajectories associated with the gain of carbapenemase genes (10). They also show that systematic genome sequencing of CP-*Ec* and on a larger scale, CPE isolates, irrespective of their clinical or resistance status, is able to provide useful information not only on the circulation of MDR lineages but also on the propagation of resistance genes through horizontal gene transfer.

## MATERIALS AND METHODS

### Isolate collection and sequencing.

CP-*Ec* isolates analyzed in this study were collected by the F-NRC, mainly between the years 2012 and 2015. Twenty-two additional isolates we received before the creation of the F-NRC in 2012 were included in the analysis. Information on these isolates such as the year of isolation, region and department in France, and summary of their genomic features are reported in Table S1 in the supplemental material.

### Whole-genome sequencing and analyses.

DNA was extracted by using the Qiagen Blood and Tissue DNA easy kit. Sequencing libraries were constructed by using the Nextera XT kit (Illumina) following the manufacturer’s instructions and sequenced with the Illumina HiSeq2500 or NextSeq500. FastQ files were trimmed for adaptors and low-quality bases (setting the minimum base quality threshold to 25) with the Cutadapt fork Atropos (33). *De novo* assemblies were generated from the trimmed reads with SPAdes v3.12.0 (34), using k-mer sizes of 51, 71, 81, and 91, the coverage cutoff option was set to “auto” and the “careful” option was activated. QUAST v2.2 (35) was used to assess the assembly quality, and contigs shorter than 500 bp were filtered out for phylogenetic analyses. The ST38 isolates CNR65D6 and CNR85I8 were sequenced to completion by using the long-read Pacbio technology. PacBio reads were assembled with the RS_HGAP_Assembly.3 protocol from the SMRT analysis toolkit v2.3 (36) and with Canu (37), polished with Quiver (36), and manually corrected by mapping Illumina reads using Breseq (38). Assembled genomes were annotated with Prokka v1.9 (39). To analyze the F-NRC CP-*Ec* isolates belonging to the main ST recovered during the analysis in a more global context, their genome sequences were combined to the assembled genome sequences from the same ST retrieved from the NCBI database (July 2019) and in some STs from EnteroBase (http://enterobase.warwick.ac.uk/, April 2021). Retrieved genomes were annotated in the same way as the genome sequences of the F-NRC isolates (Table S2).

To generate a core genome phylogeny tree of all isolates, the best reference genome was first selected among a set of 18 genomes analyzed by Touchon et al. (40) using the software refRank (41) and three subsets of 100 randomly selected sequences from our study as input. This led to the selection of the genome sequence of strain MG1655-K12 (NC_000913.3) as the reference for read mapping and SNP identification. Sequence reads for the 17 reference genomes (40) as well as for the genome sequence of Escherichia fergusonii strain ATCC 35469 (NC_011740.1), used as an outgroup, were simulated with ART (42). Trimmed sequencing reads were mapped against the MG1655-K12 genome with BWA-MEM algorithm of the BWA v0.7.4 package (43). For SNP calling, the Genome Analysis Toolkit (GATK) v3.6.0 (44) was used with the following criteria, a minimum depth coverage (DP) of 10, a quality by depth (QD) bigger than 2, a fisher strain bias (FS) below 60, a root mean square of the mapping quality (MQ) above 40, and the mapping quality rank sum test (MQRankSum) and the read position rank sum test (ReadPosRankSum) greater than −12.5 and −8, respectively. A maximum likelihood tree was estimated with RAxML v8.28 (45) using core genome SNPs after removing positions in the accessory genome identified with the filter_BSR_variome.py script from the LS-BSR pipeline (46).

For STs with more than 10 CP-*Ec* isolates from the F-NRC, a core genome alignment was generated with Parsnp (47), by using a finished genome sequence as reference. A closely related isolate outside the ST lineage was selected from the global phylogenetic tree, including all F-NRC isolates and used as an outgroup to root the ST phylogenetic trees. Maximum likelihood (ML) trees were generated with RAxML v8.28 (45) after removing regions of recombination with Gubbins (48). All graphic representations were performed by using ITOL (49).

### MLST type, phylogroup, resistance gene, and plasmid replicon identification.

Sequence type was assigned to each assembly through a python script that relies on BLAST (10). Antibiotic resistance genes (ARGs) and mutations in *gyrA*, *parC*, and *parE* quinolone resistance determining regions (QRDRs) were identified with Resfinder 4.0 and PointFinder (50) run locally, respectively. The scripts and database (retrieved 4 January 2021) were downloaded from the repositories of the Centre for Genomic Epidemiology (https://bitbucket.org/genomicepidemiology/). The identified ARGs were manually reviewed to eliminate potential redundant ARGs predicted at the same genomic position. *mdf*(A) that is present in E. coli core genome was not taken into account. Phylogroups were assigned by using EzClermont (51) run locally. Plasmid replicons were identified with plasmidfinder run on each assembly (14). *ftsI*, *ompC*, and *ompF* coding sequences (CDS) and *ompF* promoter sequences were identified by using BlastN. Translated or nucleotide sequences were clustered by cd-hit (cd-hit-v4.8.1) with an amino acid (FtsI, OmpC, OmpF) or nucleotide sequence (*ompF* promoter) identity threshold of 1. Sequences of each cluster were aligned to detect mutations in regions of interest: 4-amino-acid insertions between P333 and Y334 and E349K and I532L mutations (FtsI), mutation modifying charge in L3 constriction loop, R195L mutation, nonsense or frameshift mutations or OmpC sequence clustering with ST-38 OmpC sequences (OmpC), nonsense mutations (OmpF) or mutations affecting one of the OmpR boxes: mutation −46T/C in OmpR-F3 box and mutation −75ΔT in OmpR-F2 box (*ompF* promoter).

### Determination of *bla*_OXA-48_ insertion site in ST38 clades 1, 2, 3, and 4.

The genome sequences of GCA_005886035.1 (CP040390.1), CNR65D6, GCA_004759025.1 (CP038505.1), and CNR85I8 were chosen as representative genomes of clusters 1, 3, 2, and 4, respectively, and aligned, by using BlastN, against the sequence of GCA_001900295.1 (CP010116.1), an ST38 isolate that does not express a carbapenemase and whose genome sequence was complete. The regions surrounding the *bla*_OXA-48_ insertion sites in each clade were more precisely aligned against the corresponding sequences of other representative genomes by using Easyfig (Easyfig_mac_2.1) (52) (see Fig. S3 in the supplemental material). To perform a BLAST analysis of insertion sites by using contigs generated from Illumina sequences, smaller sequences were chosen as queries as following: nucleotides (nt) 1240133 to 1240934, nt 964173 to 965072, nt 419599 to 421606, nt 4206783 to 4207690 from GCA_001900295.1 to screen for native sequences corresponding to the four *bla*_OXA-48_ insertion sites in G1, G2, G3, and G4 clades and sequences nt 4460332 to 4460759 from CP040390.1, nt 4763987 to 4764506 from CNR65D6, nt 100784 to 102087 from CP038505.1, and nt 1206426 to 1206985 from CNR85I8 to screen for the integration site plus IS*1* insertion as occurring in G1, G2, G3, and G4 clades, respectively.

### Statistical analysis.

Pearson’s chi-squared tests were performed by using standard libraries contained within the R statistics package (http://www.R-project.org).

### Availability of data.

All sequence data have been deposited at DDBJ/EMBL/GenBank (BioProject PRJEB46636) and BioSample identifiers for the Illumina sequence data are listed in Table S1. Complete genome sequences of CNR65D6 and CNR85I8 and the long-read sequencing data have been deposited at DDBJ/EMBL/GenBank with the accession numbers OU701452.1 (PacBio reads, ERR6414227) and OU701449.1 (PacBio reads, ERR6414228), respectively.
