# Supplementary figures and images for: Specificities and Commonalities of Carbapenemase-Producing Escherichia coli Isolated in France from 2012 to 2015
Source: mSystems. 2022 Jan 11;7(1):e01169-21. doi: 10.1128/msystems.01169-21 (PMC8751382; doi:10.1128/msystems.01169-21)

A

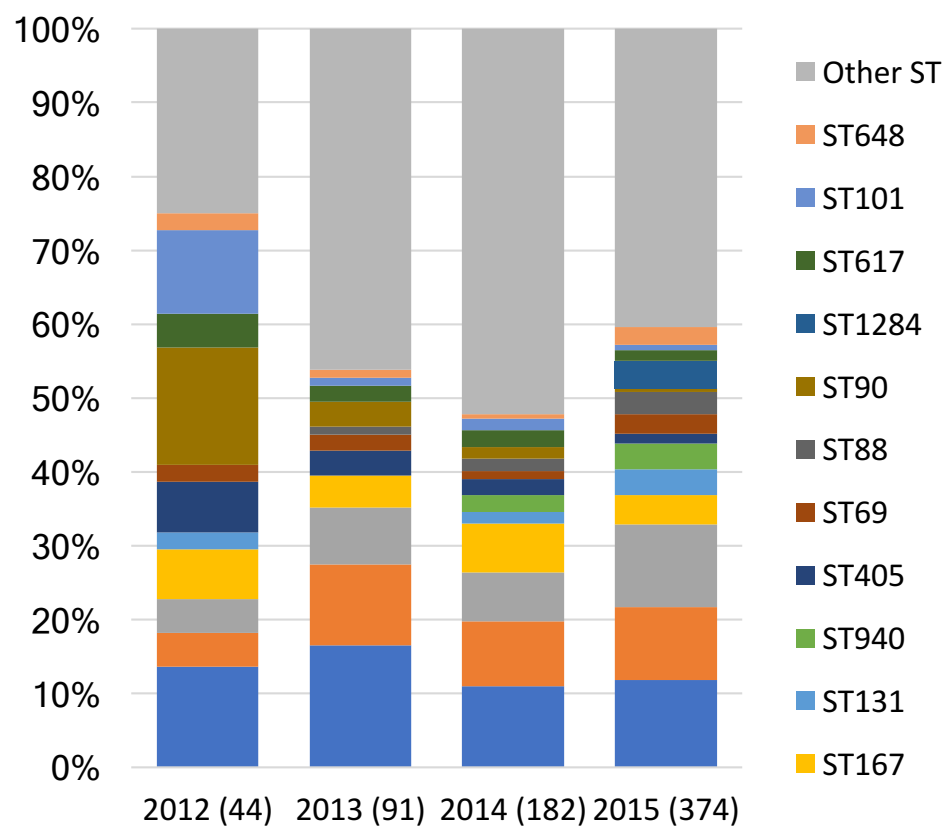

B

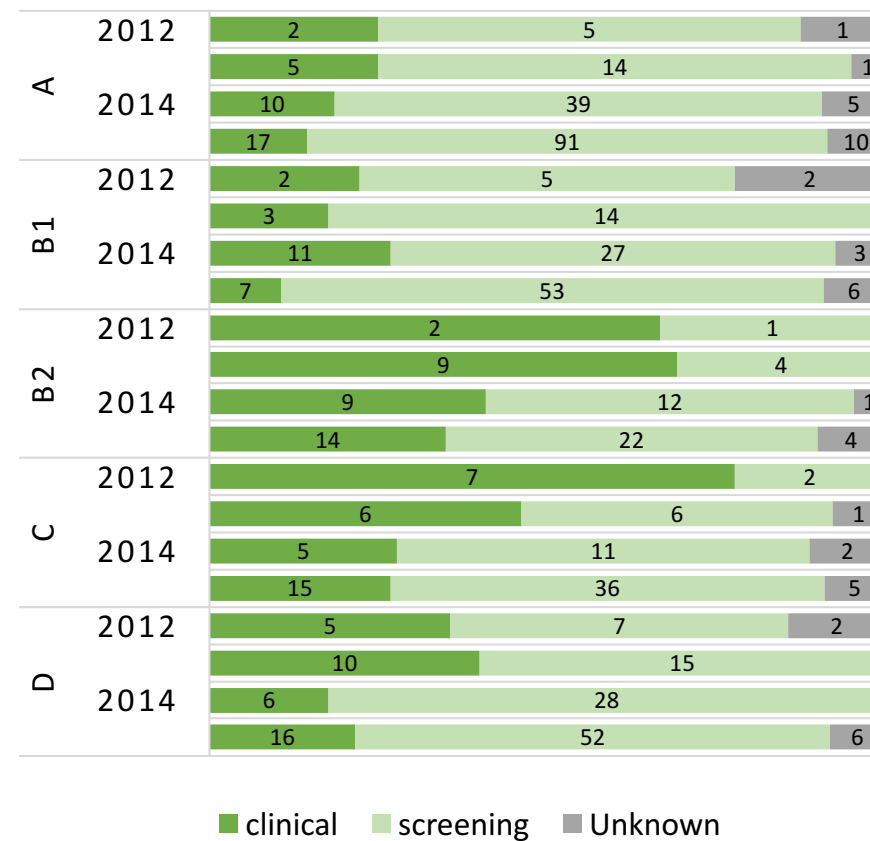

Figure S1. Per-year analysis of the origin of the isolates received by the F-NRC.

Supplement: FIG S1 [file msystems.01169-21-sf001.pdf]

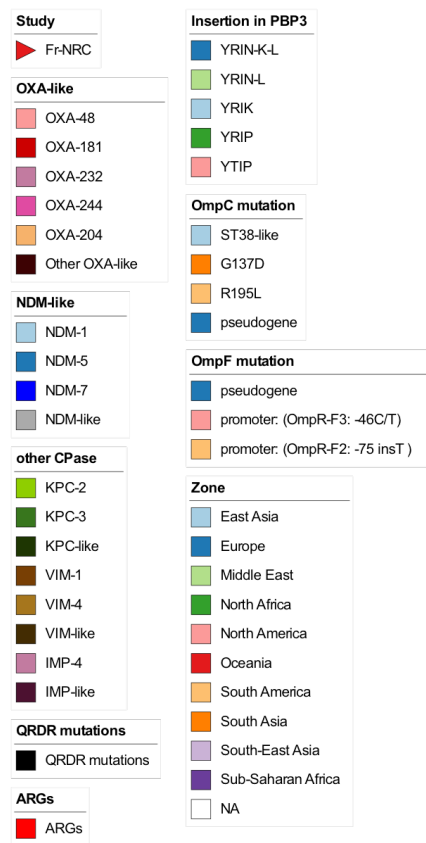

A

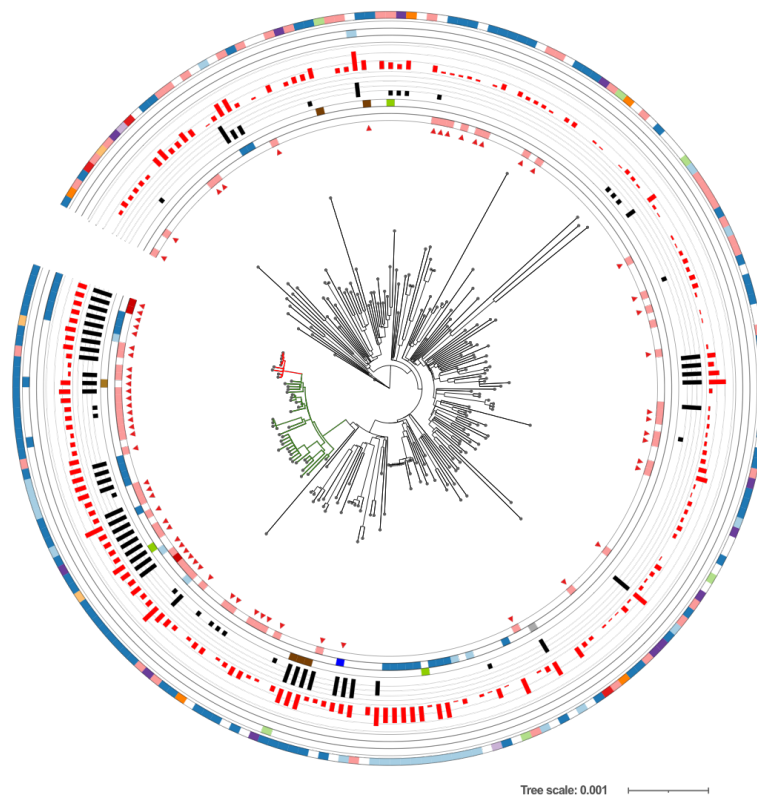

B

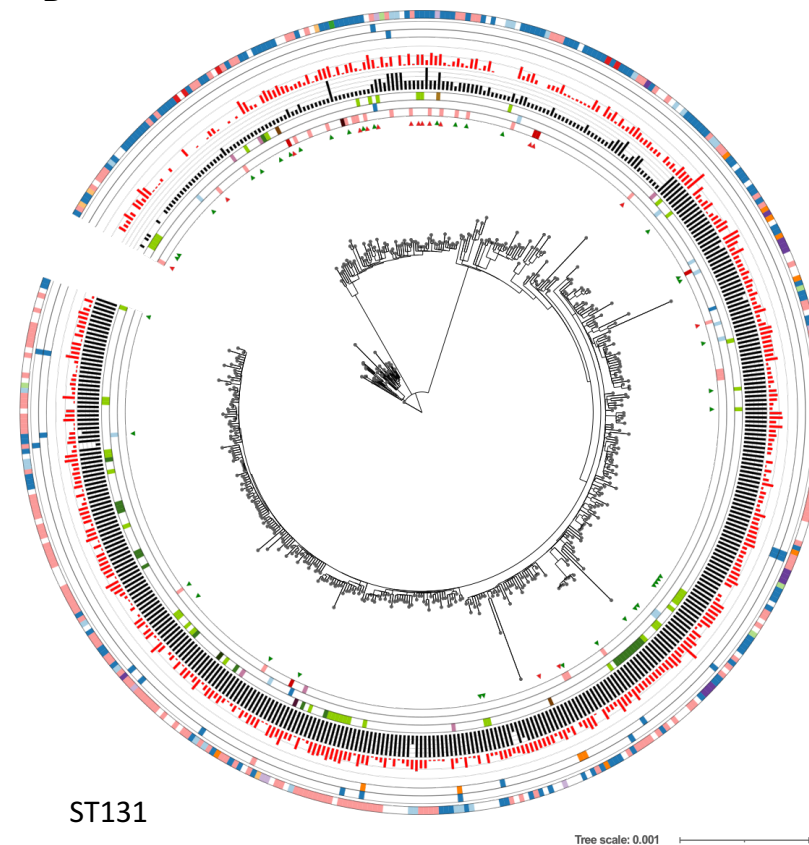

**Figure S3. Core genome phylogenies of ST10 and ST131 isolates.**

Supplement: FIG S3 [file msystems.01169-21-sf003.pdf]

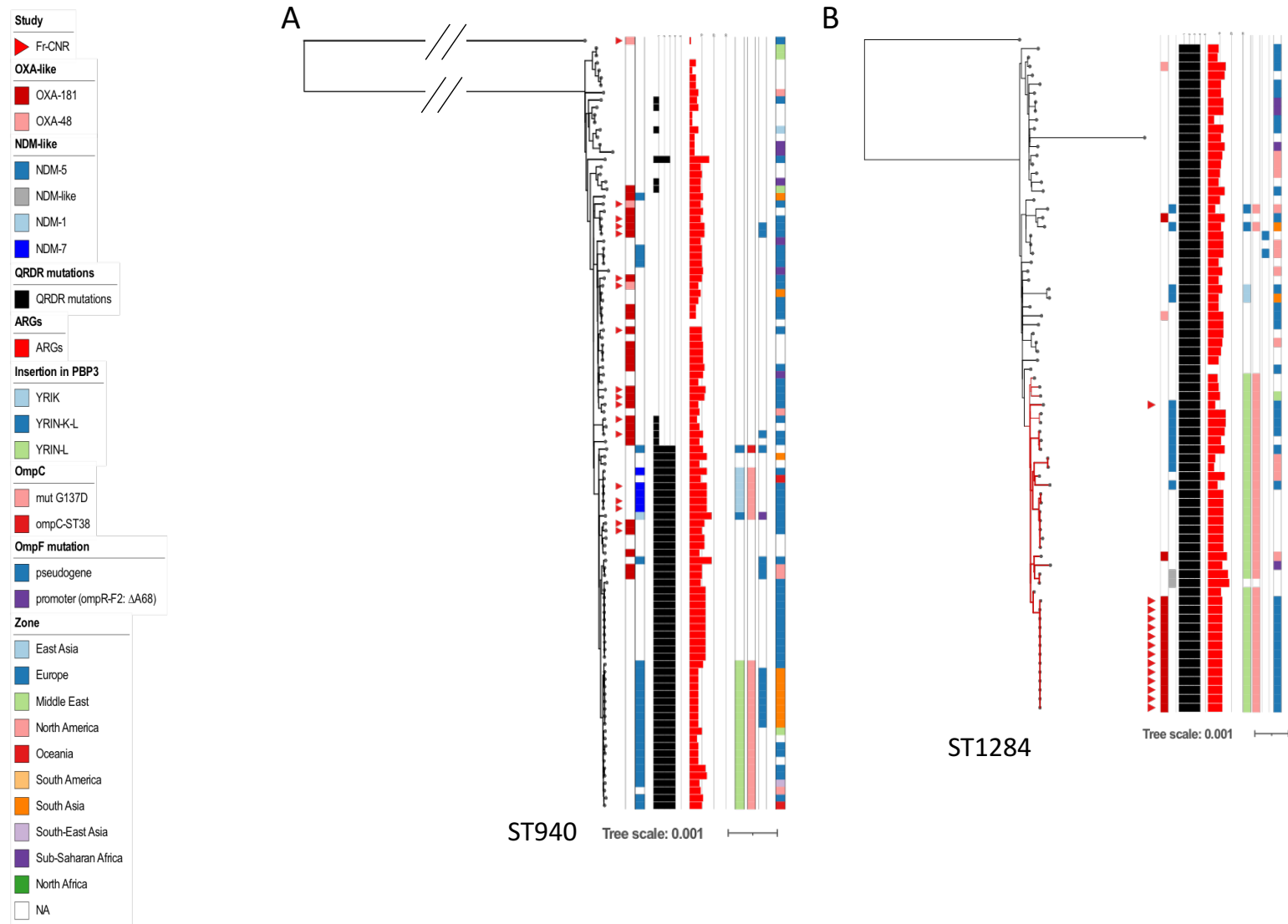

**Figure S4. Core genome phylogeny of ST940 and ST1284 isolates. A:**

Supplement: FIG S4 [file msystems.01169-21-sf004.pdf]

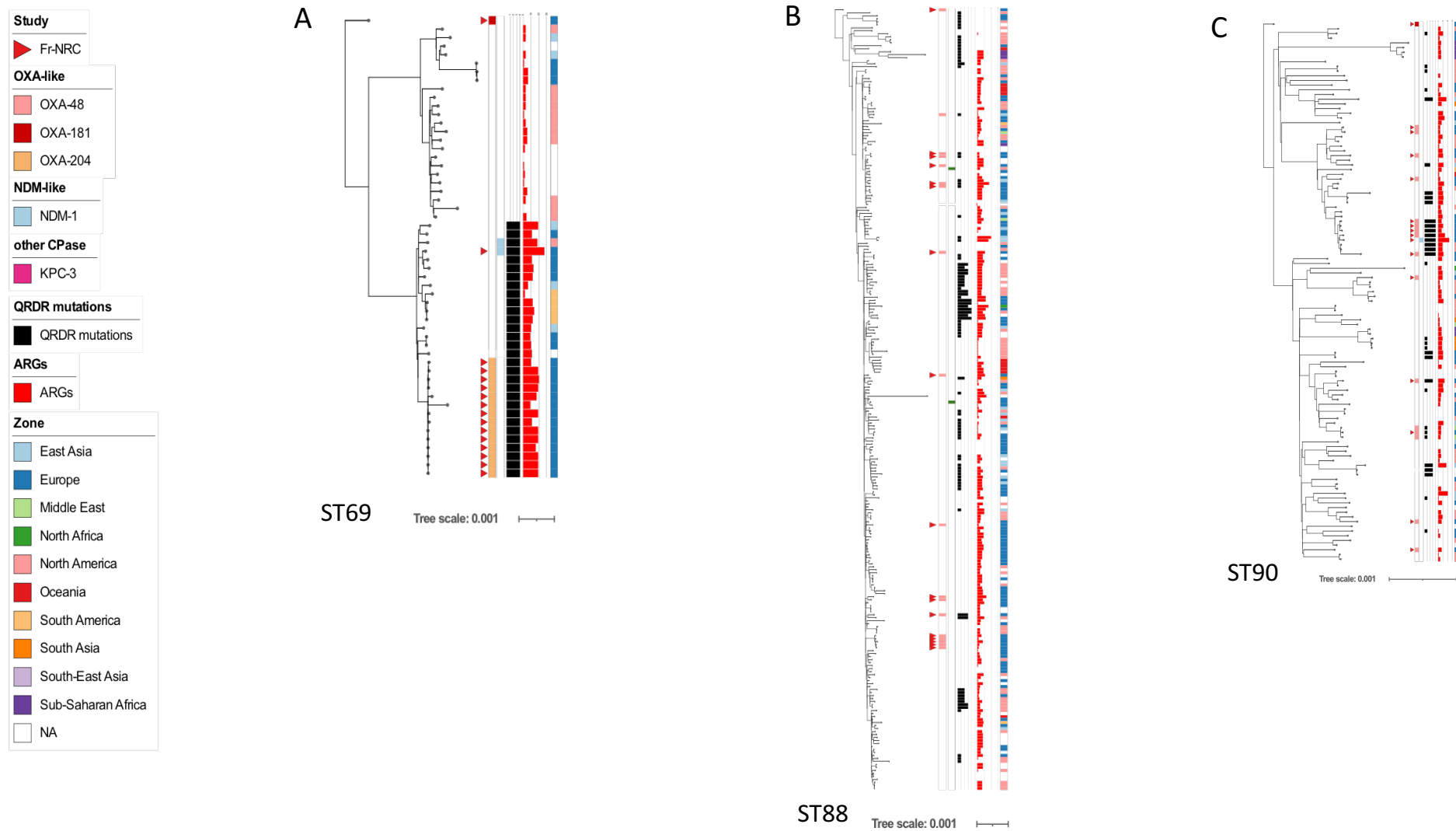

**Figure S5. Core genome phylogenies of ST69, ST88 and ST90. isolates.**

Supplement: FIG S5 [file msystems.01169-21-sf005.pdf]

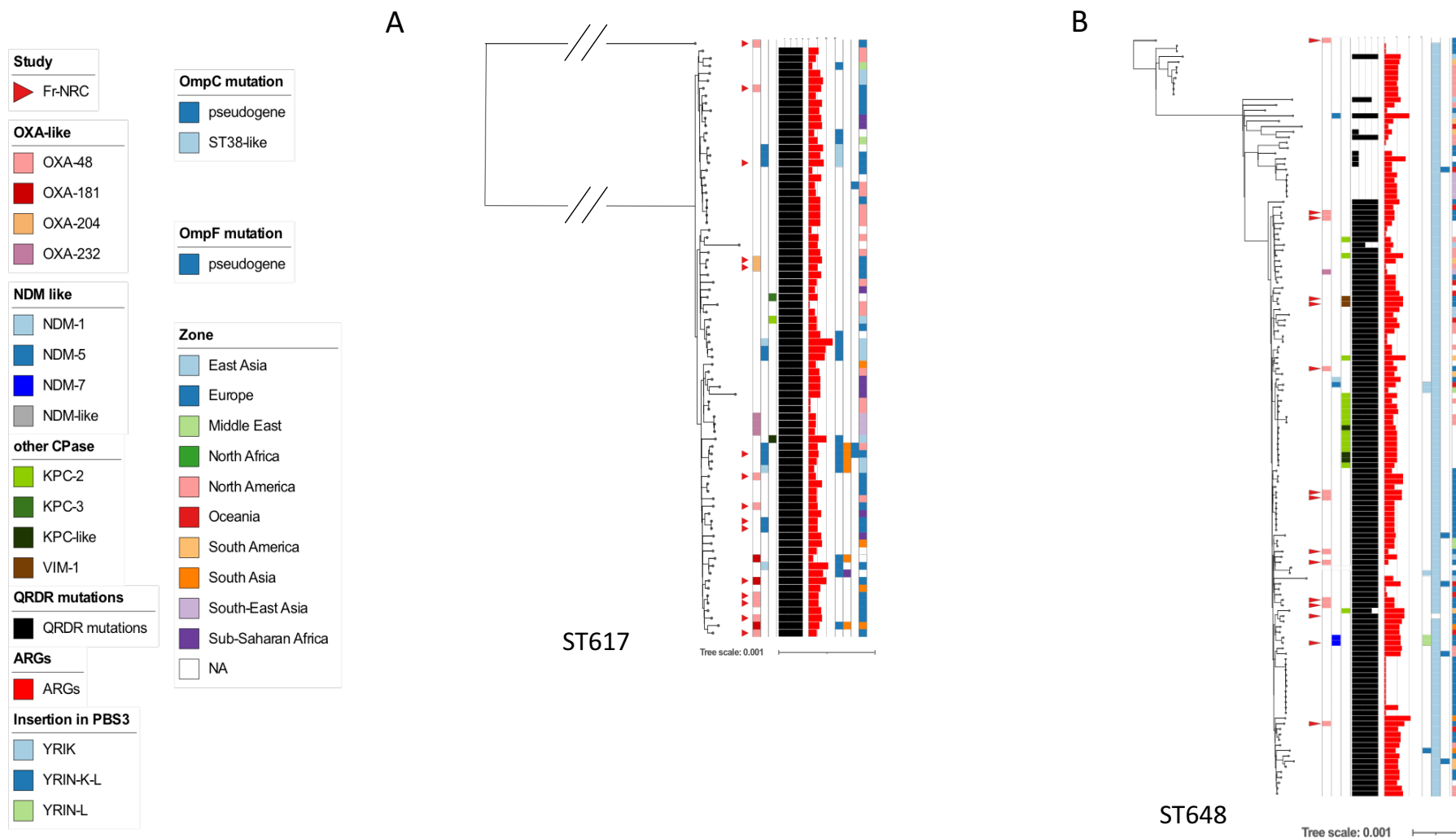

Figure S6. Core genome phylogeny of ST617 and ST648 isolates.

Supplement: FIG S6 [file msystems.01169-21-sf006.pdf]
