# Supplementary material for: Specificities and Commonalities of Carbapenemase-Producing Escherichia coli Isolated in France from 2012 to 2015
Source: mSystems. 2022 Jan 11;7(1):e01169-21. doi: 10.1128/msystems.01169-21 (PMC8751382; doi:10.1128/msystems.01169-21)
Supplement: FIG S2 [file msystems.01169-21-sf002.pdf]

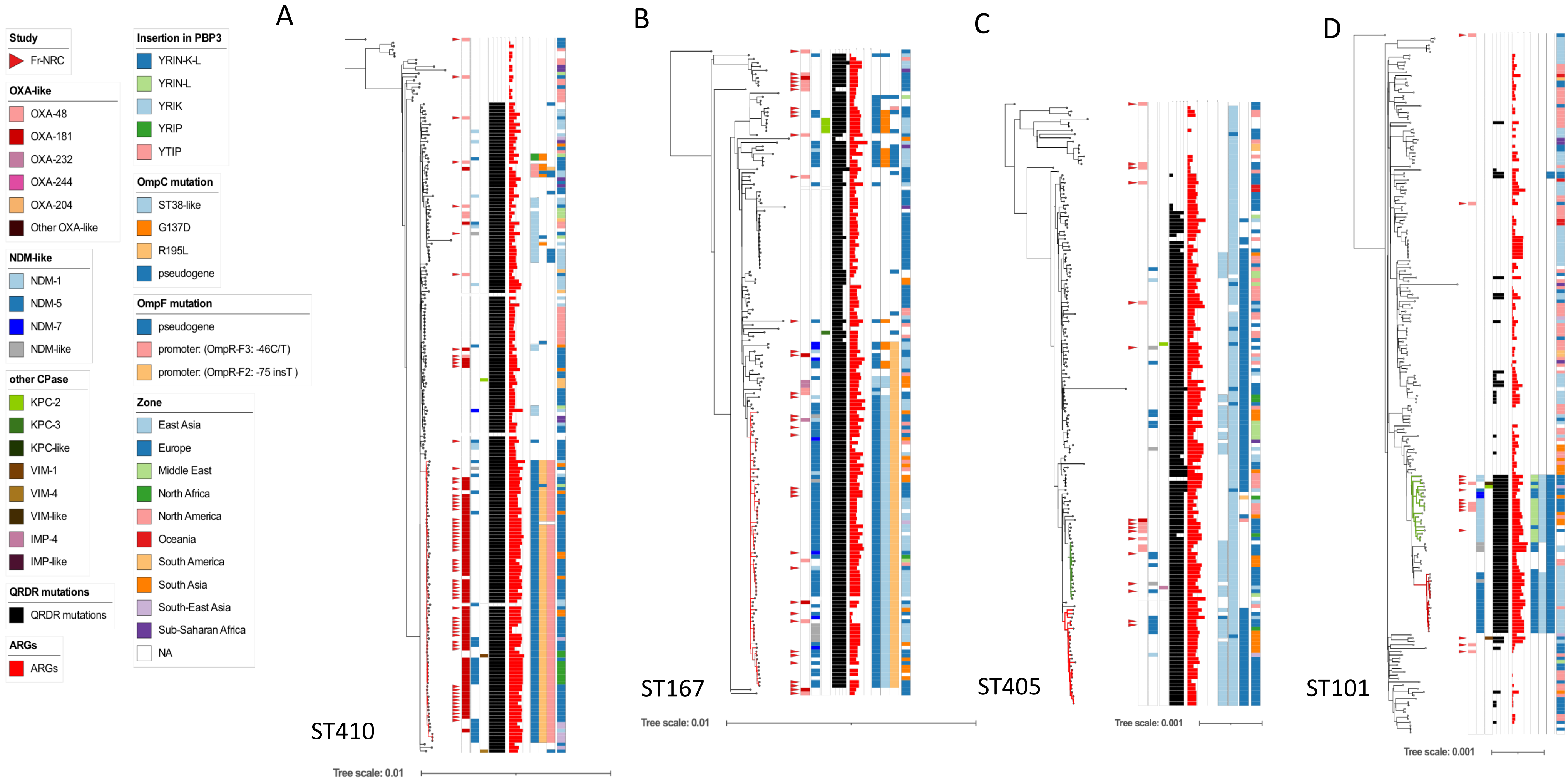

Figure S2. Core genome phylogenies of the main ST characterized by clades disseminating internationally.
